# Heterogeneity of the rice microbial community of the Chinese centuries‐old Honghe Hani rice terraces system

**DOI:** 10.1111/1462-2920.15114

**Published:** 2020-07-07

**Authors:** Pascal Alonso, Laurence Blondin, Pierre Gladieux, Frédéric Mahé, Hervé Sanguin, Romain Ferdinand, Denis Filloux, Eric Desmarais, Frédérique Cerqueira, Baihui Jin, Huichuan Huang, Xiahong He, Jean‐Benoit Morel, Darren P. Martin, Philippe Roumagnac, Christian Vernière

**Affiliations:** ^1^ CIRAD, BGPI Montpellier France; ^2^ BGPI, INRAE, CIRAD, Institut Agro, Univ Montpellier Montpellier France; ^3^ INRA, BGPI Montpellier France; ^4^ ISEM, CNRS, University of Montpellier, IRD, EPHE Montpellier France; ^5^ State Key Laboratory for Conservation and Utilization of Bio‐Resources in Yunnan Yunnan Agricultural University Kunming 650201 China; ^6^ Southwest Forestry University Kunming China; ^7^ Computational Biology Group Institute of Infectious Diseases and Molecular Medicine, University of Cape Town Cape Town 4579 South Africa

## Abstract

The Honghe Hani rice terraces system (HHRTS) is a traditional rice cultivation system where Hani people cultivate remarkably diverse rice varieties. Recent introductions of modern rice varieties to the HHRTS have significantly increased the severity of rice diseases within the terraces. Here, we determine the impacts of these recent introductions on the composition of the rice‐associated microbial communities. We confirm that the HHRTS contains a range of both traditional HHRTS landraces and introduced modern rice varieties and find differences between the microbial communities of these two groups. However, this introduction of modern rice varieties has not strongly impacted the overall diversity of the HHRTS rice microbial community. Furthermore, we find that the rice varieties (i.e. groups of closely related genotypes) have significantly structured the rice microbial community composition (accounting for 15%–22% of the variance) and that the core microbial community of HHRTS rice plants represents less than 3.3% of all the microbial taxa identified. Collectively, our study suggests a highly diverse HHRTS rice holobiont (host with its associated microbes) where the diversity of rice hosts mirrors the diversity of their microbial communities. Further studies will be needed to better determine how such changes might impact the sustainability of the HHRTS.

## Introduction

During and since the Green Revolution, governments and agricultural stakeholders all around the world have promoted the widespread introduction to cropping systems of modern high‐yielding crop varieties. By contributing to the simplification and homogenization of cropping systems this trend has likely also inadvertently contributed to increases in the occurrence of various crop diseases (Keesing *et al*., 2006; Stuckenbrock and McDonald, 2008; Roossinck and Garcia‐Arenal, 2015; Bernardo *et al*., 2018). Nevertheless, there are a number of Globally Important Agricultural Heritage Systems (GIAHS) within which landraces (traditional varieties) are still cultivated (FAO, http://www.fao.org/giahs/en/). Although many of these GIAHSs were left largely unchanged by the Green Revolution, some, such as the Honghe Hani rice terraces system (HHRTS), have more recently experienced both the introduction of modern high‐yielding rice varieties and an increase in chemical usage (Yang *et al*., 2017; Dedeurwaerdere and Hannachi, 2019).

The HHRTS, which was recently listed as a World Cultural Heritage Site (UNESCO, 2013), is a renowned rice terrace landscape where remarkably diverse rice varieties have been cultivated for over 1300 years by the Hani people (Jiao *et al*., 2012). The HHRTS consists of a collection of unique man‐made vertically structured ecological landscapes comprising cascading terraced rice fields sandwiched between forests and villages at the top, and a river at the bottom (Cui *et al*., 2008; Yang *et al*., 2017). Hundreds of years of rice varietal selection within the HHRTS has yielded at least 195 local rice landraces (*Oryza sativa*) and 47 wild rice landraces (*Oryza rufipogon* and *Oryza nivara*) (Jiao *et al*., 2012). These diverse rice landraces are grown by each Hani household in complex heterogeneous mosaics, in narrow paddy fields averaging ~150 m^2^ within each of which only one rice landrace is cultivated.

While increased diversification of rice varieties has likely reduced the severity of rice‐associated diseases (Zhu *et al*., 2000), it is usually hypothesized that the structure of the HHRTS has likely also limited the occurrence and spread of diseases within the system. For example, the spread of *Pyricularia oryzae*, the causal agent of rice blast, is apparently hampered by the high diversity of basal and effector‐triggered immune responses that are displayed by the *japonica* and *indica* rice varieties commonly cultivated within the HHRTS (Liao *et al*., 2016).

However, a recent socio‐economic survey has revealed a significant increase in the severity of diseases occurring within HHRTS fields where the government‐promoted ‘HongYang’ improved rice variety has been cultivated (Dedeurwaerdere and Hannachi, 2019). Increased use of this improved variety since 2010 may, for example, account for increases over the past decade in the prevalence within the HHRTS of Southern rice black‐streaked dwarf virus (SRBSDV) (Alonso *et al*., 2019).

Besides the role played in plant health by intrinsic plant mechanisms, plant‐associated microbial communities are also likely to be directly or indirectly involved in plant health and plant development (Berendsen *et al*., 2012; Hacquard *et al*., 2017; Hassani *et al*., 2019; Vannier *et al*., 2019). It is now widely accepted that the structuring of plant microbiota is controlled by plant‐specific factors (Vorholt, 2012; Bulgarelli *et al*., 2013; Reinhold‐Hurek *et al*., 2015; Hamonts *et al*., 2018) and that plant genotype may further impact microbial communities (Bulgarelli *et al*., 2015; Sapkota *et al*., 2015; Wagner *et al*., 2016). Specifically, plant domestication or selection has possibly contributed to a small but significant change in plant microbial communities (Redford *et al*., 2010; Peiffer and Ley, 2013; Bouffaud *et al*., 2014; Ofek‐Lalzar *et al*., 2014; Bulgarelli *et al*., 2015; Edwards *et al*., 2015). The HHRTS is therefore a good potential candidate for determining the impacts of cultivating modern rice varieties on aspects of traditional rice cultivation systems: impacts such as the composition of their associated microbial communities. Overall, modern varieties have low genetic diversity due to the recurrent selection for traits contributing to, among other things, plant yield, rice quality, and resistance to biotic and abiotic stresses (Meyer and Purugganan, 2013). This genetic simplification has often been implicated in reducing the bacterial and fungal diversity in the rhizosphere and phyllosphere of modern cultivated crop varieties relative to that found in the rhizosphere and phyllosphere of wild species and landraces (reviewed in Cordovez *et al*., 2019). For example, modern cultivated pea and broad bean varieties have less promiscuous interactions with symbionts than do their wild relatives (Mutch and Young, 2004). Similarly, the bacterial and fungal diversity in the rhizosphere and phyllosphere of uncultivated agave species is higher than that of cultivated agave (Coleman‐Derr *et al*., 2016). Small but significant domestication effects have also been noted when comparing the root microbial communities of wild and modern varieties of barley (Bulgarelli *et al*., 2015; Szoboszlay *et al*., 2015). Two potential exceptions to this trend are modern lettuce and sunflower varieties, which harbour degrees of rhizobacterial diversity that are similar to, or in some cases higher than, those of their uncultivated relatives (Cardinale *et al*., 2015; Leff *et al*., 2017).

The microbial community associated with the rice rhizosphere has been the most intensively investigated (Edwards *et al*., 2015; Wang *et al*., 2016; Edwards *et al*., 2018; Moronta‐Barrios *et al*., 2018; Ding *et al*., 2019). Specifically, several studies have focused on the comparison of the rhizosphere or seed‐associated microbial communities of wild *Oryza*, rice landraces and modern rice varieties (Shenton *et al*., 2016; Shi *et al*., 2019; Kim *et al*., 2020). For instance, Shenton and colleagues (2016) have shown that the rhizosphere bacterial community of wild *Oryza* differed substantially from that of cultivated rice in terms of both species richness and composition (Shenton *et al*., 2016). Interestingly, this study also revealed that landraces had a rhizosphere bacterial community composition that was intermediate between that of wild *Oryza* and cultivated rice varieties (Shenton *et al*., 2016). It is also apparent that fungal communities differ more substantially than bacterial communities between the seed or rhizosphere microbial communities of cultivated varieties and wild *Oryza* (Shi *et al*., 2019; Kim *et al*., 2020).

Here, we hypothesized that the introduction of modern rice varieties to the HHRTS may have impacted rice plant microbial communities within the HHRTS. We initially determined the degree of rice genetic diversity within the paddy fields of a single HHRTS village, Malizhai that has adopted a mixed landrace/modern variety system. Then, we compared the diversity and composition of the microbial communities of introduced modern rice varieties and HHRTS landraces.

## Results and discussion

### Both landraces and modern rice varieties are grown in the Malizhai HHRTS

Malizhai is a village broadly representative of the HHRTS throughout the Hani region (Jiao *et al*., 2012). To obtain a detailed understanding of rice genetic diversity within the Malizhai HHRTS, we sampled 19 rice paddies, each in a 2 km^2^ area of Malizhai. While 11 of the rice paddies from which samples were collected were referred to as ‘traditional varieties’ by the Malizhai farmers, eight were referred to as ‘modern varieties’. We performed a genotyping by sequencing (GBS) analysis of the 19 sampled rice paddies (hereafter referred to as our Malizhai GBS dataset) and compared these to a reference dataset containing whole‐genome re‐sequencing data produced by the 3000 Rice Genomes Project (Wang *et al*., 2018) from nine rice accessions randomly selected from among four representative subpopulations of the *indica* rice subspecies (subpopulations XI‐1A, XI‐1B, XI‐2 and XI‐3).

**Fig 1:**
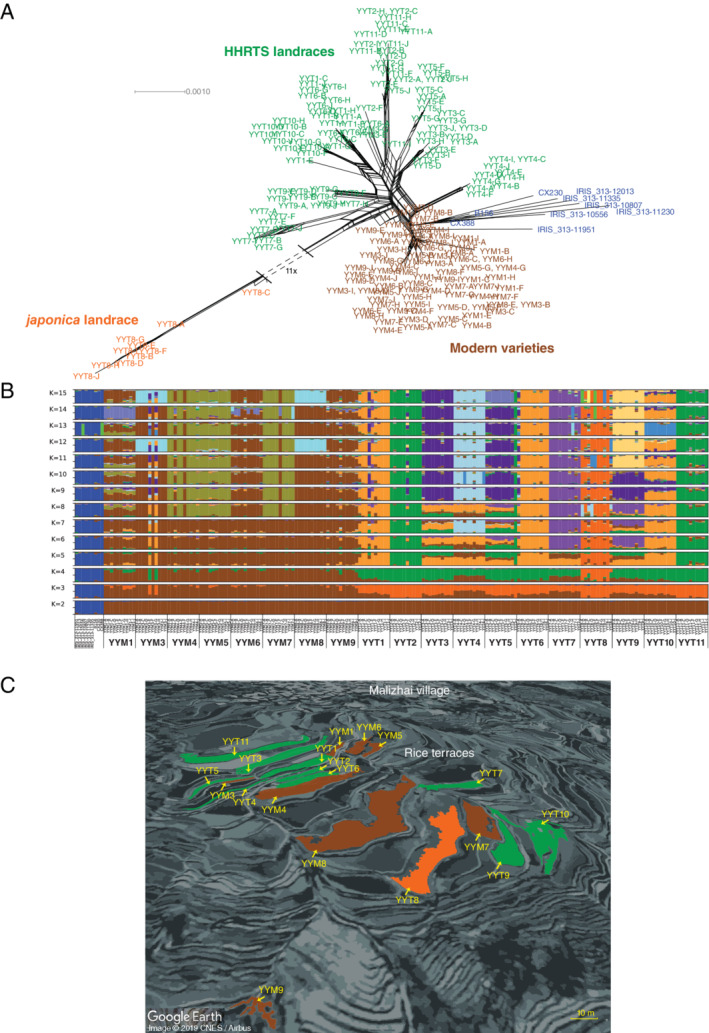
A. Neighbour‐Net split decomposition network indicating the relationships between rice accessions based on 10 028 analysed SNPs. Plant samples assigned to the ‘HHRTS landraces’, the ‘modern rice varieties’, the *japonica* subspecies and representative subset of *indica* subgroups from the 3000 Rice Genomes Project are labelled in green, brown, orange and blue respectively. B. Ancestry proportions inferred using sNMF for models with *K* = 2 to *K* = 15 ancestral populations. Each accession is represented by a vertical bar divided into K segments of different colours, representing proportions of ancestry in K ancestral populations for a single accession, with colours corresponding to ancestral populations. C. Map of the Malizhai rice terraces and location of the rice fields that were collected in 2016. Terraces were HHRTS landraces, modern rice varieties and japonica varieties, which are coloured in green, brown and orange respectively.

This GBS comparison revealed that only one of the groups of Malizhai rice genotypes belonged to the *japonica* rice subspecies with the remaining 18 group of rice genotypes belonging to the *indica* rice subspecies (Fig. 1A, B). Whereas 10 of the 18 group of Malizhai *indica* genotypes were identified by farmers as being ‘traditional varieties’ and will hereafter be referred to as the ‘group of HHRTS landraces’, the other eight genotypes were identified by farmers as being ‘modern varieties’ and will hereafter be referred to as the ‘group of modern rice’. Our inference of population subdivision by partitioning genotypes into K ancestral populations, based both on the cross‐entropy criterion (Fig. S1) and visual analysis of clustering patterns (Fig. 1B), revealed that the model with *K* = 4 clusters captured most of the structure in the data. Hence, at *K* = 4, besides the *indica* rice genotypes from the 3000 Rice Genomes Project (coloured in blue, Fig. 1B), three groups from the Malizhai HHRTS were distinguishable, including a group of *japonica* genotypes (coloured in orange, Fig. 1B), the group of HHRTS landraces (coloured in green, Fig. 1B), and the group of modern varieties (coloured in brown, Fig. 1B). Our GBS analysis confirmed that the rice genotypes are heterogeneously distributed in the Malizhai HHRTS forming a mosaic landscape of modern genotypes and landraces (Fig. 1C). The eight groups of genotypes representing the group of modern varieties included commercial modern hybrid F1 varieties that are widely and intensively cultivated in the rice‐growing plains of China (Table 1). Nucleotide diversity was slightly lower within the group of modern varieties (*π* = 8.05 × 10^−4^) than that of the HHRTS landraces group (*π* = 9.43 × 10^−4^).

**Table 1:** Genotyping of rice varieties from the Yuanyang rice terraces of China and assignment of rice varieties to three rice genetic groups (‘HHRTS landraces group’, ‘Modern rice group’ and *Oryza sativa* subspecies *japonica*).

Sampling field	Rice genetic group	Rice variety name(given by the farmers)	Number of samples
YYT1	HHRTS landraces group (HongYang)	Chepugu	9
YYT2	HHRTS landraces group	Lubaigu	10
YYT3	HHRTS landraces group	Zaogu	10
YYT4	HHRTS landraces group	Nuogu	10
YYT5	HHRTS landraces group	Epugu	10
YYT6	HHRTS landraces group (HongYang)	Chepugu	9
YYT7	HHRTS landraces group	Jianshuigu	10
YYT8	*Oryza sativa* ssp. *japonica*	Honglueduolu	10
YYT9	HHRTS landraces group	Nuogu	10
YYT10	HHRTS landraces group (HongYang)	Jinpinggu	10
YYT11	HHRTS landraces group	Luhonggu	10
YYM1	Modern rice group	Mingliangyou 527	10
YYM3	Modern rice group	Mingliangyou 528	8
YYM4	Modern rice group	Hefeng 177	10
YYM5	Modern rice group	Zhongyou 177	10
YYM6	Modern rice group	Liangyou 2186	10
YYM7	Modern rice group	Guofeng 1	10
YYM8	Modern rice group	Liangyou 725	10
YYM9	Modern rice group	Liangyou 2161	10

The model with *K* = 8 clusters also revealed patterns of clustering that seem biologically sensible. At *K* = 8, the group of modern varieties could be further split into two subgroups, referred to as the modern rice subgroup1 (samples from fields YYM1, YYM3, YYM6, YYM8 and YYM9, Fig. 1B) and the modern rice subgroup2 (samples from fields YYM4, YYM5 and YYM7, Fig. 1B). Unexpectedly, but in accordance with farmer assignments, the government‐promoted HongYang varieties grown in three fields (YYT1, YYT6 and YYT10; Table 1) that were believed to be ‘modern improved varieties’ clearly share a recent ancestry with the HHRTS landraces (Fig. 1A). This implies that HongYang is based on a traditional landrace that likely originated from the group of HHRTS landraces (Dedeurwaerdere and Hannachi, 2019). Clustering patterns at *K* = 8 also revealed that the group of HHRTS *indica* landraces can be further subdivided into four subgroups, which are referred to as HHRTS landraces subgroup1 (YYT1, YYT6 and YYT10, i.e. HongYang varieties), HHRTS landraces subgroup2 (YYT2, YYT11), HHRTS landraces subgroup3 (YYT3, YYT5) and HHRTS landraces subgroup4 (YYT7, YYT9) (Fig. 1B).

We have thus found that rice varieties grown in the Malizhai HHRTS fall into two main genetic groups; one including newly introduced commercial Hybrid F1 modern varieties and the other including several traditional or improved (HongYang) varieties. This confirms that changes in cultural practices in the Malizhai HHRTS, and likely in other villages as well, have resulted in a shift in the genetic makeup of cultivated rice in the terraces. We also find evidence that further replacement of HHRTS landraces by modern varieties could slightly reduce the over‐all genetic diversity of rice within the HHRTS.

### The microbial communities of rice roots and stems are highly divergent

Having characterized the genetic diversity of rice grown in the Malizhai HHRTS, we next sought to characterize the bacterial and fungal components of the microbial communities of sampled rice plants. Our samples included the microbes living at the surface and within plant tissue. Based on the analysis of GBS and whole‐genome resequencing data, we split the sampled *indica* rice plants into a group of modern varieties (including 78 plants from eight different modern varieties) and a group of HHRTS landraces (including 98 plants from 10 different traditional or improved HHRTS varieties). For consistency, we excluded plants of the YYT8 *japonica* rice subspecies (Table 1) from this analysis. We then examined the stem and root microbial communities of plants belonging to the HHRTS landrace and modern variety groups.

Metabarcoding of bacterial communities produced after the bioinformatics processing 120 666 high‐quality reads from the root compartment and 92 400 from the stem compartment respectively corresponding to 325 and 91 Operational taxonomic units (OTUs) with each OTU representing over 50% prevalence per rice variety (Table S1). For the fungal communities, we obtained 30 858 high‐quality reads for the root compartment and 21 750 high‐quality reads for the stem for the root compartment respectively representing 110 and 105 OTUs with each OTU representing over 50% prevalence per rice variety (Table S1). Details of the number of sequences and OTUs recovered after each step of the bioinformatics processing are available in Table S1. Given that too few samples from the field YYT4 yielded enough high‐quality reads to reach the rarefaction thresholds, this variety was further excluded from the group of HHRTS landraces in all subsequent analyses. Data for the group of HHRTS landraces were drawn from 88 plant samples belonging to nine different rice traditional varieties. The OTUs tables are available in Tables S2 and S3.

Root bacterial communities were significantly richer than those of the stem (Fig. S2A) and the structure and composition of these bacterial communities were also significantly different (Fig. S2C). Additionally, while the α diversity indices (measured by the richness index and Shannon index) of fungal root and stem communities were not significantly different (Fig. S2B), the fungal community compositions were significantly different between the stem and root compartments, albeit to a lower degree than that observed for the bacterial communities (Fig. S2C/D). These results remain consistent with previous observations in other species that the strongest determinant of the microbial community compositions is the compartment from which these are drawn (Knief *et al*., 2012; Lundberg *et al*., 2012; Coleman‐Derr *et al*., 2016).

### Wide inter‐individual variation in α diversity of microbial communities associated with HHRTS rice plants

**Fig 2:**
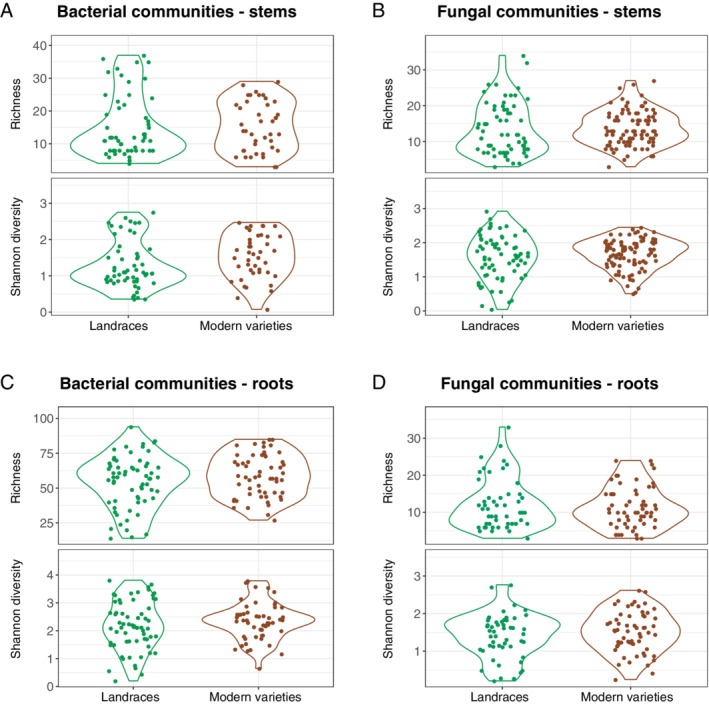
Violin plots of rice microbial α diversity (richness and Shannon diversity indexes) across HHRTS landraces and modern rice varieties for (A) the rice stem bacterial communities and (B) the rice stem fungal communities as well as (C) the rice root bacterial communities and (D) the rice root fungal communities. *P*‐values of Tukey HSD tests were all >0.05 between landrace and modern communities whatever the community (bacteria or fungi) and the diversity indexes.

The root and stem microbial community of HHRTS rice plants were subsequently studied separately to detect potential impacts on these of the shifting composition of rice plant populations that is currently occurring in the HHRTS (landraces vs. modern varieties). Alpha diversity indices were not significantly different between modern rice varieties and HHRTS landraces (Fig. 2) when considering either bacterial or fungal communities in either the stem or root samples. Additionally, the six‐subgroups partitioning revealed a low variability in alpha diversity indices among HHRTS rice subgroups even if the indices of α diversity between landraces subgroups 3 and 4 were significantly different (Fig. S3). It should be stressed, however, that the α diversity indices estimated across landraces and modern varieties, or for each of the six subgroups of landraces and modern varieties were highly variable (Fig. 2 and Fig. S3), suggesting that factors other than rice genotype may be shaping the richness of microbial communities at the individual rice plant level. Such wide inter‐individual variations in α diversity are reminiscent of the inter‐individual variability of microbial communities observed previously in human saliva. This variability in saliva has been interpreted as a possible consequence of the human mouth being exposed to highly variable external (food) and internal (digestion exchanges) environments, and being subjected to a wide variety of oral hygiene regimes (Hall *et al*., 2017; Verma *et al*., 2018). Accordingly, our results suggest that individual rice plants within a field may have encountered during their lifetimes a variety of physical and chemical environmental conditions that have left an imprint on the composition of their associated microbial communities. The impacts of heterogeneous micro‐environments on rice‐associated microbial community structures may be particularly pertinent to flooded rice systems in general (Fernández‐Valiente and Quesada, 2004; Cui *et al*., 2008; Xie *et al*., 2011; Jiao *et al*., 2012), and specifically to the HHRTS flooded rice system, where micro‐environment variability could conceivably arise for a multitude of different reasons including (i) the high densities at which rice is planted; (ii) the compactness of the terraces; (iii) the low depth of the floodwaters within the terraces; (iv) the variability of ancillary food production systems that are co‐located in the terraces which can include ducks, fish, frogs, and snails; and (v) erratic increases in the levels of suspended soil particles in the floodwaters arising as a consequence of periodic disturbances of silt by labourers and water buffalos within the paddies.

### Microbial community structure of modern rice varieties and HHRTS landraces are slightly different

**Fig 3:**
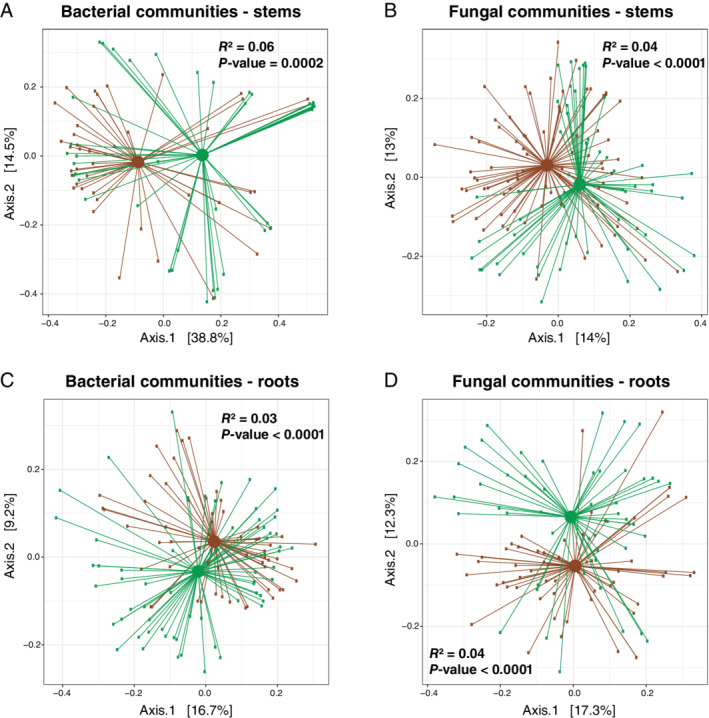
Principal coordinates analysis (PCoA) plots based on unweighted UniFrac distances of (A) rice stem bacterial communities and (B) rice stem fungal communities as well as (C) rice root bacterial communities and (D) rice root fungal communities. Communities from the HHRTS landraces and from the modern rice varieties are labelled in green and brown respectively. Axes represent the two dimensions explaining the greatest proportion of variance in the communities for each analysis. Permutational multivariate analysis of variance (PERMANOVA) results are indicated (*R* ^2^ and the *P*‐value).

We further examined differences between the microbial community compositions associated with modern rice varieties and those associated with HHRTS landraces. We first calculated the ß‐diversity and used a permutational multivariate analysis of variance (PERMANOVA) of microbial communities between modern rice varieties and HHRTS landraces for both stem and root samples using phylogeny‐based UniFrac distances either weighted or unweighted by the abundance of OTUs. These PERMANOVA analyses revealed a slight (*R* ^2^ values ranged from 0.03 to 0.06), but significant effect of variety type for both stem and root bacterial and fungal communities using the unweighted UniFrac distances (Fig. 3). This finding supports the hypothesis that the introduction of modern rice varieties in the HHRTS could have caused a slight modification in the structure of rice‐associated microbial communities. This slight effect is consistent with findings from recent studies on other plant species (Emmett *et al*., 2017; Hamonts *et al*., 2018; Compant *et al*., 2019). In contrast, the quantitative weighted UniFrac distances did not support a difference between the structures of HHRTS landrace microbial communities and the modern variety microbial communities (Fig. S4). This result suggests that the slight microbial variations observed between both rice groups in the Malizhai HHRTS are primarily driven by differences in the distributions of rare taxa between the microbial community associated with the HHRTS landrace and modern variety groups.

### Rice variety is a key factor of the structure of the microbial community in Malizhai HHRTS

**Fig 4:**
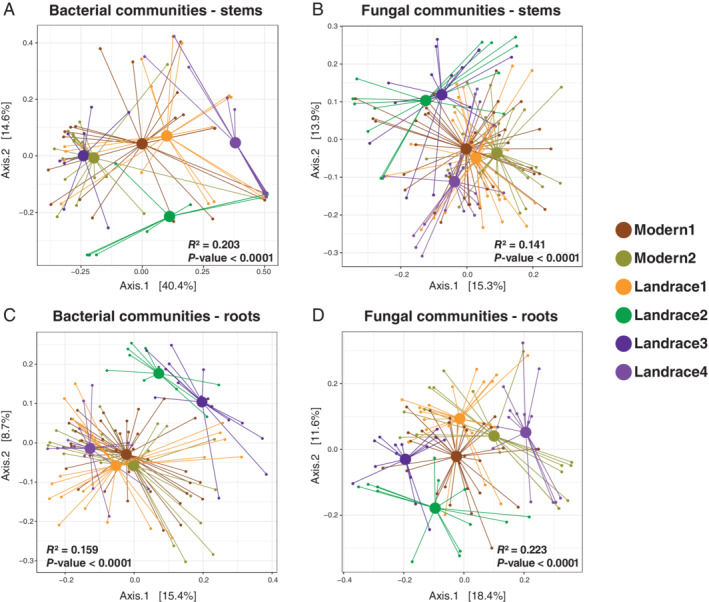
Principal coordinate analysis (PCoA) plots based on unweighted UniFrac distances of (A) rice stem bacterial communities, (B) rice stem fungal communities, (C) rice root bacterial communities and (D) rice root fungal communities. Communities associated with the six subgroups of HHRTS rice varieties are labelled in orange (HHRTS landraces subgroup1), green (HHRTS landraces subgroup2), blue (HHRTS landraces subgroup3), purple (HHRTS landraces subgroup4), brown (modern subgroup1) and olive green (modern subgroup2) respectively. Axes represent the two dimensions explaining the greatest proportion of variance in the communities for each analysis. Permutational multivariate analysis of variance (PERMANOVA) results are indicated (*R* ^2^ and the *P*‐values).

We examined differences between the microbial community compositions associated with the six rice subgroups of landraces and modern varieties that were sampled in 2016 in the Malizhai HHRTS and identified using clustering algorithms assuming *K* = 8 clusters (Fig. 4). The PERMANOVA analysis revealed a significant effect of the rice subgroup (*R* ^2^ values ranged from 0.14 to 0.22, *P* < 0.0001) for both stem and root bacterial and fungal communities using the unweighted UniFrac distances (Fig. 4). This result suggests that rice subgroups, each composed of related rice varieties, play a role in structuring the HHRTS microbial communities. This finding is in line with results from another study that revealed that genotypic differences in rice had a significant effect on root‐associated microbial communities (Edwards *et al*., 2015). Principal Coordinate Analyses (PCoA) based on unweighted UniFrac distances showed that bacterial communities from both stems and roots, and fungal communities from the stems of the modern subgroups and landrace subgroup1 (corresponding to the governmental‐promoted HongYang varieties) were more similar to each other and farther from the three other landraces subgroups (Fig. 4). The pairwise PERMANOVA analysis using the unweighted UniFrac distances further confirmed that the bacterial communities from roots were not significantly different between both modern rice subgroups and between the modern rice subgroup2 and the landrace subgroup1 (Table S4). This result suggests that varietal improvement for higher yields or pest resistance that was derived either from improved local landraces (HongYang varieties, landrace subgroup1) or from exogenous rice varieties (modern high‐yielding rice hybrids) has led to a partial homogenization of microbial communities among the improved varieties grown in the Malizhai HHRTS. This convergence in microbial communities can be explained by shared selected features of improved rice varieties such as their root morphology or their root exudation (or other traits) that have induced the assembly of similar microbial communities in the Malizhai HHRTS context (Szoboszlay *et al*., 2015; Perez‐Jaramillo *et al*., 2018; Cordovez *et al*., 2019). Based on these results, we tested whether partitioning the Malizhai HHRTS varieties into ‘improved varieties’ (including modern and HongYang varieties) and ‘traditional landraces’ (including landraces subgroups 2, 3 and 4) would have revealed a higher degree of population structuring in the Malizhai HHRTS microbial communities. The PERMANOVA analysis revealed a slight (*R* ^2^ values ranged from 0.04 to 0.05), but significant effect of variety type for both stem and root bacterial and fungal communities using the unweighted UniFrac distances, suggesting that the ‘varietal improvement’ factor explain less the structure of microbial communities than the ‘rice genotype’ factor.

We further compared the relative abundance of individual OTUs between the six rice subgroups of landraces and modern varieties using Wilcoxon signed‐rank tests. We then used the Metacoder approach, which relies on a taxonomic tree‐based visualization (called a heat tree), to illustrate whether the relative abundance of microbial OTUs differed significantly between the six rice subgroups (Figs [Link], [Link], [Link] and S8). These analyses highlighted 243 OTUs with significantly different (with a *P*‐value threshold <0.05) degrees of relative abundance between the six rice subgroups (Tables S5 and S6). Only two fungal OTUs of the 243 OTUs were more abundant in stems of all four rice subgroups of traditional and improved HHRTS varieties than in both modern rice subgroups (Table S6), suggesting a reduced core microbial community of rice varieties cultivated in the Malizhai HHRTS.

### Phylogenetic distances between the microbial community and rice genotypes were correlated

Given that we found differences between the microbial communities of modern rice varieties and HHRTS landraces, we investigated whether more closely related genotypes tend to have more similar microbial communities. We conducted Mantel tests using the unweighted UniFrac distances of OTUs and genetic distances between rice genotypes. All four of the unweighted UniFrac distance matrices of microbial communities were significantly correlated with the genetic distances between the rice genotypes they are associated with (Table S7). This result is consistent with previous studies focusing on wild and modern rice varieties (Shenton *et al*., 2016; Kim *et al*., 2020), maize and other Poaceae (Bouffaud *et al*., 2014) suggesting that the evolutionary history of poaceous crop plants has had an impact on the evolutionary history of their microbial communities.

### The core microbial community associated with HHRTS rice plants encompassed a small number of taxa

We hypothesized that the ‘core’ microbial community in the stem and root of Malizhai HHRTS rice plants is limited in size. To test this hypothesis, we considered OTUs only present in at least 80% of the stem and root samples from plants belonging to the 18 *indica* varieties (including all the modern varieties and HHRTS landraces), with no consideration of the relative abundance of the taxa. The core stem microbial community consisted of just two fungal OTUs and one bacterial OTU and the core root microbial community consisted of just two fungal OTUs and three bacterial OTUs (Table 2). We further considered separately the stem and root core microbial communities of the modern rice varieties and HHRTS landraces. The core stem microbial community of modern rice varieties consisted of two fungal OTUs and the HHRTS landraces core stem microbial community included these two‐same fungal OTUs and one additional bacterial OTU (Table 2 and Table S8). Whereas the core root microbial community of the modern rice varieties consisted of six bacterial OTUs and one fungal OTU that of the HHRTS landraces consisted of two bacterial OTUs and one fungal OTU (Table 2 and Table S8). Consequently, the core microbial community defined by our criteria ranged from 0.7% to 3.3% of the total number of taxa identified within and among the HHRTS landraces and modern rice varieties (Table 2). These low percentages are consistent with another study that identified a core microbial community constituted of 1.5% of the total OTUs associated with rice plants sampled from three Californian rice fields (Edwards *et al*., 2015). The relative abundances of the ‘core’ OTUs associated with the Malizhai HHRTS rice varieties accounted for between 0.7% and 26.4% of the total number of assigned sequencing reads (see relative abundances in Table S8). The core microbial community, therefore, consisted of a combination of rare and abundant OTUs (Table S8). Besides revealing a high heterogeneity of HHRTS microbial community, our study also indicates that the HHRTS rice holobiont [host with its associated microbes that can potentially affect the phenotypes of rice plants (Vandenkoornhuyse *et al*., 2015; Theis *et al*., 2016)] is highly diverse with microbial communities mirroring the diversity their rice hosts.

**Table 2:** The proportion of core reads and core OTUs within and among the rice genetic groups.

Community	Plant compartment	Genotype group	Number of core reads	Total number of reads	Proportion of core reads	Number of core OTUs	Total number of OTUs	Proportion of core OTUs
Bacterial	Stem	Modern + HHRTS landraces	18 703	101 850	18.3	1	91	1.0
		Modern	0	44 100	0.0	0	63	0.0
		HHRTS landraces	11 233	57 750	19.5	1	81	1.2
	Root	Modern + HHRTS landraces	41 838	120 666	34.7	3	325	0.9
		Modern	28 417	56 784	50.0	6	240	2.5
		HHRTS landraces	23 336	63 882	36.5	2	288	0.7
Fungal	Stem	Modern + HHRTS landraces	13 737	45 240	30.4	2	110	1.8
		Modern	8185	20 590	39.8	2	60	3.3
		HHRTS landraces	5501	24 650	22.3	2	91	2.2
	Root	Modern + HHRTS landraces	6534	30 858	21.2	2	105	1.9
		Modern	2729	13 344	20.4	1	71	1.4
		HHRTS landraces	1898	17 514	10.8	1	79	1.3

### Towards disentangling the biotic and abiotic factors affecting the Malizhai HHRTS rice microbial community

Our results show that the population structure of Malizhai HHRTS rice varieties is one of the factors that impact the community structure of associated microbes. However, even if the genetic makeup of rice hosts explained the largest proportion of variance in the composition of microbial communities in Malizhai HHRTS (15%–22%), other factors not covered by our study may also explain the observed patterns of microbial diversity. Indeed, the small sampling area selected (<2 km^2^) was more environmentally heterogeneous than initially envisioned. It is known that plant root microbial communities are influenced by soil cultivation histories and agricultural practices (Peiffer *et al*., 2013; Li *et al*., 2019). Hence, the turnover of rice varieties in HHRTS fields and other characteristics of the flooded terraces—such as the use of living animal fertilizers (ducks, snails, fish and cattle)—may also explain the heterogeneity of the microbial communities of different rice plants (Xie *et al*., 2011; Jiao *et al*., 2012). Precise information pertaining to the soil compositions or fertilizer usage history of our sampling sites is not available. However, previous surveys of farming practices in the portion of the HHRTS that we studied revealed that farmers employ a variety of cropping practices and frequently alter the diverse set of rice varieties that they use (Dedeurwaerdere and Hannachi, 2019).

Another factor that could have contributed to environmental heterogeneity is the presence of rice pathogens. Several recent studies have indicated both that the impact of microbial communities on the course and outcome of host diseases can be substantial, and that host diseases can reciprocally have a large impact on microbial community compositions (Berendsen *et al*., 2012; Ritpitakphong *et al*., 2016; Koskella *et al*., 2017; Zhang *et al*., 2018; Vannier *et al*., 2019). We have recently shown that the SRBSDV was prevalent in 2016 in the Malizhai HHRTS and that 23 of the 166 rice plants examined in the present study were infected by SRBSDV (Alonso *et al*., 2019). We, therefore, examined the impact of SRBSDV infections on the compositions of microbial communities but no significant difference was observed between the microbial communities of SRBSDV‐infected rice plants and those of SRBSDV‐uninfected plants (Table S9). Interestingly, several of the HHRTS landraces and modern rice varieties had no SRBSDV infected plants. If these landraces and varieties are either resistant or tolerant to SRBSDV (Alonso *et al*., 2019), it is most probable that this resistance would be attributable to host genetic factors, i.e. host resistance, rather than to their respective microbial communities. SRBSDV is transmitted by a flying insect such that root microbial communities are less likely to play a significant role in SRBSDV transmission than in the transmission of soil‐borne rice pathogens.

Finally, in addition to deterministic factors, like host genotype that did explain the largest proportion of variance in the compositions of rice microbial communities, and other factors not covered by this study, we cannot rule out that stochastic processes could have also influenced microbial community compositions. Indeed, stochastic processes might account for the high variability in microbial community compositions that were observed both between plants belonging to the same rice genotypes and between plants sampled from the same HHRTS paddy fields (Zhou and Ning, 2017). Stochasticity could for instance arise during the colonization of plants by microbes wherever early arriving taxa modify the surface or within‐plant niches, making these niches more or less suitable for later‐arriving species (Maignien *et al*., 2014). Therefore, besides better disentangling the roles that biotic and abiotic factors play in modifying the microbial community of HHRTS rice plants, further studies will be needed to determine the impacts over time of stochastic processes on dynamic changes in rice microbial community compositions.

## Materials and methods

### Study area and sampling site

The village of Malizhai is located near the town of Xinjie in the Honghe Hani Yi Autonomous Prefecture (Yunnan province, China). This village has recently adopted a mixed landrace/modern variety system but, prior to 2010 was representative of the cultural landscape of the Hani rice terraces (Jiao *et al*., 2012). The sampling site in Malizhai covered an area of approximately 2 km^2^ (N23°07′55.04″ E102°46′03.95″) at an altitude ranging between 1570 and 1608 m.

### Plant sampling

Nineteen small Malizhai paddy fields were sampled in July 2016, including 11 fields cultivated with HHRTS landraces and eight fields cultivated with “modern rice varieties. Ten plants were collected per field, regardless of the presence of disease symptoms. It is noteworthy that at the time of the sampling survey disease pressure was very low and a very few symptoms were visible on rice plants throughout the Malizhai terraces. Roots, stems and leaves were separately collected and rinsed with water in order to remove the attached soil. All samples were individually stored at 4°C in a mobile fridge. Within 24 h, all samples were dried in the presence of CaCl_2_ until DNA extraction.

### Plant and microbial DNA extractions

Plant DNA extractions and genotyping‐by‐sequencing GBS were carried out on 30 mg of dried individual leaf samples as described previously (Alonso *et al*., 2019). We performed microbial DNA extractions on each stem sample with both epiphyte and endophyte microorganisms and on each root sample with its rhizoplane (root surface) and endophyte microorganisms. In total, 30 mg of the 190 sample rice stems and the 190 sample rice roots were individually frozen in liquid nitrogen then ground with bead beating (two steel beads, diameter 0.1 cm and one ceramic bead, diameter 0.5 cm) using a FastPrep‐24 5G System (MP Biomedicals ‐ Fisher Scientific) for 2 × 30 s at 6 ms^−1^. Total genomic DNA was extracted from the resulting powder using the NucleoMag Plant Kit (Macherey–Nagel, Germany) and KingFisher Flex Purification System (ThermoFisher Scientific, MA, USA), following the manufacturer's instructions.

### Analysis of rice genotyping‐by‐sequencing data

GBS reads for rice landraces and whole‐genome re‐sequencing data for nine randomly selected individuals from four representative subpopulations of *indica* (XI‐1A, XI‐1B, XI‐2 and XI‐3) described in the 3000 Rice Genomes Project (Wang *et al*., 2018) were aligned against the Nipponbare rice reference genome (MSU7) using Bowtie2.3.5 (Langmead *et al*., 2009; Langmead and Salzberg, 2012; Wang *et al*., 2018; Langmead *et al*., 2019). The GBS raw sequence read data are available from the NCBI Sequence Read Archive, under the BioProject ID number PRJNA573048. SNP‐calling using bcftools mpileup (options ‐‐max‐depth 500 ‐a DP) was carried out independently for the 3000 Rice Genomes Project dataset and the GBS dataset (https://www.sanger.ac.uk/science/tools/samtools-bcftools-htslib). For the GBS dataset, sites with either AN ≤ 300 or MQ ≤ 20 or which were tagged as ‘LowQual’ were removed using bcftools filter (INFO/AN ≤ 300; INFO/MQ ≤ 20) and bash (grep ‐v ‘LowQual’). Genotypes with DP ≤ 5 were converted to missing data using vcftools (‐‐minDP 5). For the 3000 Rice Genomes Project dataset, sites with DP ≤ 5 or MQ ≤ 20 were filtered out using bcftools filter (INFO/DP ≤ 5; INFO/MQ ≤ 20). The VCF files for the 3000 Rice Genomes Project and GBS accessions were merged using bcftools merge. The merged dataset included 143 611 sites with ≤50% missing data and 10 028 sites with ≤10% missing data. We used the dataset with ≤10% missing data for analyses of genealogical relationships among genotypes.

We constructed a neighbour‐network using SplitsTree 4.13, to visualize evolutionary relationships between the *indica* rice genotypes while taking the possibility of recombination or incomplete lineage sorting into account (Huson and Bryant, 2006). We used the program sNMF (http://membres-timc.imag.fr/Olivier.Francois/snmf/index.htm) to test the dataset with ≤50% missing data for evidence of population subdivision by partitioning genotypes into K ancestral populations and estimating individual ancestry coefficients in the K populations (Frichot *et al*., 2014). We ran sNMF for a number of clusters K‐values ranging from 1 to 15, and for each K we performed 10 replicates. We used the Greedy algorithm in CLUMPP 1.1.2 (http://web.stanford.edu/group/rosenberglab/clumpp.html) (Jakobsson and Rosenberg, 2007) to identify runs representing the same clustering solution (i.e. same mode). We randomly selected one representative of runs belonging to the major mode for graphical representation as a stacked barplot using the Matplotlib package in Python. Nucleotide diversity (*π*) was estimated using the scikit‐allel in Python (https://github.com/cggh/scikit-allel).

### PCR amplification and sequencing

The composition and diversity of rice‐associated microbial communities were characterized by applying a high‐throughput sequencing‐based protocol that targets PCR‐generated amplicons. Bacterial communities were characterized from the variable region, V3‐V4, of the 16S rRNA gene using the primers 341‐F (5′CTACGGGNGGCWGCAG 3′) and 785‐R (5′GACTACHVGGGTATCTAATCC3′) as universal primers to maximize bacterial taxonomic assignment (Thijs *et al*., 2017). We used ITS86‐F (5′GTGAATCATCGAATCTTTGAA3′) and ITS4‐R (5′ TCCTCCGCTTATTGATATGC3′) as universal primers for amplification of the fungal ITS1 region (Op De Beeck *et al*., 2014).

DNA amplification was performed by PCR in a total volume of 25 μl containing 1× GoTaq G2 DNA polymerase buffer (Promega Corporation, Madison, USA), 0.5 μM of each primer, and 0.2 μM dNTPs and 1 μl of genomic DNA. We used peptide nucleotide acid (PNA) clamps, which specifically bind to mitochondrial or chloroplast sequences to block the amplification of plant derived‐DNA. We added to the PCR mix PNA blocker oligos (PNA Bio, Thousand Oaks, CA, USA) at 0.5 μM targeting the 16S rRNA gene of plant mitochondria (PNAm: GGCAAGTGTTCTTCGGA), and chloroplasts (PNSp: GGCTCAACCCTGGACAG) (Jackrel *et al*., 2017). All amplifications were performed in a thermal cycler (Biometra, Gottingen, Germany) under the following conditions for 16S rRNA gene amplifications: an initial denaturation at 98°C for 3 min followed by 30 cycles of denaturation at 98°C for 15 s, PNA annealing at 75°C for 10 s, primers annealing at 52°C for 10 s, extension at 72°C for 30 s, and a final extension at 72°C for 10 min. ITS amplifications were performed under the following conditions: an initial denaturation at 94°C for 3 min followed by 30 cycles of denaturation at 94°C for 45 s, primers annealing at 55°C for 45 s, extension at 72°C for 2 min, and a final extension at 72°C for 10 min. Each PCR product was tagged with a combination of two different barcodes designed by a genomic platform (GenSeq, University of Sciences, UMII, Montpellier, France) that allows for the identification of 384 different PCR products loaded onto the same MiSeq flow cell. Negative controls from the extraction step and PCR reaction were sequenced with the plant samples to evaluate and exclude contaminant reads from the sample data set. All PCR products were pooled and purified, and the library was constructed and sequenced using a GenSeq platform with Illumina paired‐end 2 × 250‐bp technology and V2 chemistry.

### Sequence processing, OTU clustering, and OTU filtering

Base calling and demultiplexing of Illumina sequences were carried out using RTA v1.18.54, MCS 2.6 and bcl2fastq2.17. Paired Illumina MiSeq reads were assembled with VSEARCH v2.11.0 (Rognes *et al*., 2016) using the command fastq_mergepairs and the option fastq_allowmergestagger. Primer clipping was performed with cutadapt v1.9 (Martin, 2011) allowing a 2/3‐length partial match for forward and reverse primers. Only reads containing both primers were retained. The expected error per read was estimated with the VSEARCH command fastq_filter and the option eeout. Each sample was then dereplicated by merging identical reads using vsearch's command, derep_fulllength, and converted to FASTA format. To prepare for clustering, the samples were pooled and further dereplicated with VSEARCH. Files containing per‐read expected error values were also dereplicated to retain only the lowest expected error for each unique sequence. Clustering was performed with Swarm v3.0.0 (Mahe *et al*., 2015), using a local threshold of one difference and the fastidious option.

OTU representative sequences were then searched for evidence of chimeras with the VSEARCH command, uchime_denovo (Edgar *et al*., 2011). In parallel, representative sequences were assigned to taxa using the stampa pipeline (https://github.com/frederic-mahe/stampa/) and the ribosomal database SILVA v138 (https://www.arb-silva.de/) (Quast *et al*., 2013) for the bacterial community, and a custom version of the ITS database UNITE v8 (https://unite.ut.ee/) (Abarenkov *et al*., 2010; Koljalg *et al*., 2013) for the fungal community.

Clustering results, expected error values, taxonomic assignments and chimera detection results were used to build a raw OTU table. Up to that point, reads without primers, reads shorter than 32 nucleotides and reads with uncalled bases (‘N’) had been eliminated. To create the ‘cleaned’ OTU table, additional filters were applied to retain only non‐chimeric OTUs, OTUs with an expected error per nucleotide below 0.0002, OTUs containing more than three reads or which were found two or more samples. ITS and 16S OTUs tables obtained after the initial cleaning step (chimeric OTUs removal) can be found at https://github.com/P‐alonso/HHRTS_microbial_diversit (Yunnan_Rice_2016_16S_roots_and_stems_384_samples.OTU.filtered.table, Yunnan_Rice_2016_ITS2_roots_and_stems_384_samples.OTU.filtered.table). All 16S and ITS sequences with a higher abundance in at least one negative control than the rice samples were excluded from the final dataset. All 16S OTUs assigned to chloroplast or mitochondrial sequences were excluded. Similarly, ITS OTUs not assigned to fungal reference sequences were excluded. All codes and representative OTU sequences can be found in HTML format in Supporting information file S1. The raw data are available from the NCBI Sequence Read Archive (SRA) under BioProject ID number PRJNA573048.

### Statistical analyses of microbial community data

All statistical analyses were performed in R (http://www.R-project.org) with the Phyloseq (McMurdie and Holmes, 2013), Vegan (Oksanen *et al*., 2018), pairwiseAdonis (Martinez Arbizu, 2020) and Metacoder packages. Biological replicates corresponding to 10 plants per field were used to fix a biological reproducibility threshold in order to perform the sequencing de‐noising. Only OTUs with over 50% prevalence per paddy field were considered. Samples with less than 1000 reads for bacterial communities and less than 5000 reads for fungal communities were discarded following which the OTUs abundances were rarefied to homogenize sequencing depth.

We estimated the microbial diversity using richness and Shannon's diversity indices for α‐diversity calculations and UniFrac distances for β‐diversity calculations. To assess the relationships between plant genotypes (HHRTS landraces vs. modern rice varieties) and plant‐associated microbial community, a Tukey HSD test and a PERMANOVA were performed for the α‐diversity and the β‐diversity respectively. Differences in community composition between groups of samples were statistically evaluated by PERMANOVA using the UniFrac distance matrices. The adonis() function was used to calculate PERMANOVA with 10 000 permutations between HHRTS and modern varieties. A pairwise *post hoc* comparison was performed to evaluate the difference in community compositions across the six rice subgroups (HHRTS landraces subgroup1, HHRTS landraces subgroup2, HHRTS landraces subgroup3, HHRTS landraces subgroup4, modern subgroup1 and modern subgroup2) using the pairwise.adonis() function. Differences in community composition were assessed by PCoA based on weighted UniFrac and unweighted UniFrac distances. PCoA is an ordination method that represents pairwise (dis)similarities between samples in a low‐dimensional space, so that samples placed closer in the graph are more similar than those placed further apart (McMurdie and Holmes, 2013). R codes used in statistical analyses are provided at https://github.com/P-alonso/HHRTS_microbial_diversit/.

The Metacoder package was used to visualize differential abundances in taxa between the modern varieties and HHRTS landrace groups using the function compare_groups() among plant microbial communities on a differential heat tree. The ratio of the mean OTU abundance between landraces and modern rice varieties was calculated. For each taxon, a Wilcoxon Rank Sum test was used to test for differences between the median abundances of samples between landraces and modern rice varieties. The taxon abundance of bacterial and fungal communities was plotted on a taxonomic tree and the result of the Wilcoxon Rank Sum test with a fold‐change cutoff of 1.5 and a *P*‐value cutoff of 0.05 were used to highlight the differences in taxon abundance between HHRTS landraces and modern rice varieties. To test for significant associations between microbial community dissimilarities and the phylogenetic distances between rice plants that hosted these communities, we conducted partial Mantel tests, as implemented in the vegan package in R (Oksanen *et al*., 2018) between the unweighted UniFrac distances of microbial communities and the patristic distances of rice plants calculated with the cophenetic() function.

The core microbial taxa within the stem and root microbial communities were identified using the Microbiome R package based on a criterion of prevalence in at least 80% of the samples from the 18 *indica* varieties (including all the modern varieties and HHRTS landraces) with no criterion related to the relative abundance of the taxa, in order to consider rare but prevalent microbial taxa. A second analysis focused on stem and root microbial taxa identified from each group of rice genotypes using the same criterion. Based on this criterion, a list of core taxa was identified and their relative abundance was calculated.

## Supporting information


**File S1** Codes and bioinformatics methods used to produce OTU tables after the initial cleaning (chimeric OTUs removal) (HTML format).

**Fig. S1** Relationship between minimal cross‐entropy and number of ancestral populations (K) modelled in the sNMF analysis of population subdivision.

**Fig. S2** Violin plots of rice microbial alpha diversity (richness and Shannon diversity indexes) of (A) root and stem bacterial communities and (B) roots and stems fungal communities. P‐values of Tukey HSD tests are shown with ***P < 0.001 or not shown if P > 0.05. PCoA plots based on unweighted UniFrac distances of (C) root and stem bacterial communities and (D) root and stem fungal communities. Communities identified from stems and roots are coloured in blue and red respectively. Axes represent the two dimensions explaining the greatest proportion of variances in the communities for each analysis. Permutational multivariate analysis of variance (PERMANOVA) results are indicated (R^2^ and the P‐value).

**Fig. S3** Violin plots of rice microbial α diversity (richness and Shannon diversity indices) across the six subgroups of rice sampled in the HHRTS (HHRTS landraces subgroup1 (L1), HHRTS landraces subgroup2 (L2), HHRTS landraces subgroup3 (L3), HHRTS landraces subgroup4 (L4), modern subgroup1 (M1) and modern subgroup2 (M2)) for (A) the rice stem bacterial communities, (B) the rice stem fungal communities, (C) the rice root bacterial communities, and (D) the rice root fungal communities. Results from Tukey HSD tests are presented as letters denoting groups that are significantly different (P‐values <0.05).

**Fig. S4** PCoA plots based on weighted UniFrac distances of (A) rice stem bacterial communities and (C) rice stem fungal communities as well as (B) rice roots bacterial communities and (D) rice roots fungal communities. Communities from the HHRTS landraces and from the modern rice varieties are labelled in green and brown respectively. Axes represent the two dimensions explaining the greatest proportion of variances in the communities for each analysis. Results of a permutational multivariate analysis of variance (PERMANOVA) are indicated (R^2^ and the P‐value).

**Fig. S5** Pairwise comparison of number of reads of rice stem bacterial communities assigned to bacterial species among the six HHRTS rice subgroups. The node width indicates the number of reads assigned to each taxonomic rank level along the tree and the colour indicates the statistically significant differences in relative bacterial taxa abundance. A taxon coloured brown is more abundant in the communities in the column and a taxon coloured green is more abundant in the communities in the row.

**Fig. S6** Pairwise comparison of number of reads of rice root bacterial communities assigned to bacterial species among the six HHRTS rice subgroups. The node width indicates the number of reads assigned to each taxonomic rank level along the tree and the colour indicates the statistically significant differences in relative bacterial taxa abundance. A taxon coloured brown is more abundant in the communities in the column and a taxon coloured green is more abundant in the communities in the row.

**Fig. S7** Pairwise comparison of number of reads of rice stem fungal communities assigned to fungal species among the six HHRTS rice subgroups. The node width indicates the number of reads assigned to each taxonomic rank level along the tree and the colour indicates the statistically significant differences in relative fungal taxa abundance. A taxon coloured brown is more abundant in the communities in the column and a taxon coloured green is more abundant in the communities in the row.

**Fig. S8** Pairwise comparison of number of reads of rice root fungal communities assigned to fungal species among the six HHRTS rice subgroups. The node width indicates the number of reads assigned to each taxonomic rank level along the tree and the colour indicates the statistically significant differences in relative fungal taxa abundance. A taxon coloured brown is more abundant in the communities in the column and a taxon coloured green is more abundant in the communities in the row.

**Table S1** Abundance of reads and OTUs in bacterial and fungal community data sets for stem and root samples through the different steps of the bio‐informatics treatments.

**Table S2** Abundance of bacterial OTUs in the rice communities of modern varieties (YYM) and HHRTS landraces (YYT) obtained from root (R as last letter) or stem (T as last letter) and taxonomic assignation. Different numbers indicate different rice paddies. Cleaned data obtained after the rarefaction process.

**Table S3** Abundance of fungal OTUs in the rice communities of modern varieties (YYM) and HHRTS landraces (YYT) obtained from root (R as last letter) or stem (T as last letter) and taxonomic assignation. Different numbers indicate different rice paddies. Cleaned data obtained after the rarefaction process.

**Table S4** Pairwise permutational multivariate analysis of variance (PERMANOVA) results for bacterial and fungal communities for both stem and root samples using unweighted UniFrac distance values (10,000 permutations), R^2^ denotes the proportion of variance that could be explained by the grouping. P. adjusted corresponds to the Bonferroni correction applied to adjust the P‐value for multiple comparisons within each group. Sig significance level *P. adjusted <0.05, **P. adjusted <0.01 and NS not significant.

**Table S5** Bacterial taxa from stems and roots that are significantly differentially abundant between the six rice subgroups using Wilcoxon signed rank tests. Only, significant P‐values <0.05 are indicated.

**Table S6** Fungal taxa from stems and roots that are significantly differentially abundant between the six rice subgroups using Wilcoxon signed rank tests. Only, significant P‐values <0.05 are indicated.

**Table S7** Detection of phylogenetic signal in root‐associated and stem‐associated microbial communities. Mantel statistic based on Pearson's product–moment correlation to assess the correlation between unweighted UniFrac distances matrix of microbial community dissimilarities and the rice genetic distances (10,000 permutations), R^2^ denotes the proportions of variances that could be explained by the grouping.

**Table S8** List of the taxa that were present in more than 80% of the plant samples of each rice genetic group.

**Table S9** Permutational multivariate analysis of variance (PERMANOVA) results for the influence of infection of SRBSDV on bacterial and fungal composition using UniFrac distance values (10,000 permutations), R^2^ denotes the proportions of variances that could be explained by the grouping.
