# Supplementary material for: Heterogeneity of the rice microbial community of the Chinese centuries‐old Honghe Hani rice terraces system
Source: Environ Microbiol. 2020 Jul 7;22(8):3429–45. doi: 10.1111/1462-2920.15114 (PMC7497281; doi:10.1111/1462-2920.15114)
Supplement: Supplementary file 1 — File S1 Codes and bioinformatics methods used to produce OTU tables after the initial cleaning (chimeric OTUs removal) (HTML format). [file EMI-22-3429-s001.docx]

**Heterogeneity of the rice microbial community of the Chinese centuries-old Honghe Hani rice terraces system**

**Table of Contents**

- [1. Disclaimer](file:///Users/verniere/Documents/Publications/YYT%20rice%20microbiota/Rice%20microbiota-HHRTS/version%20soumise/Revised%20version/Revised%20Version_Last/Supporting%20information/SI_File_S1.html#org7db01de)
- [2. Check quality encoding](file:///Users/verniere/Documents/Publications/YYT%20rice%20microbiota/Rice%20microbiota-HHRTS/version%20soumise/Revised%20version/Revised%20Version_Last/Supporting%20information/SI_File_S1.html#orgd429769)
- [3. R1-R2 quality](file:///Users/verniere/Documents/Publications/YYT%20rice%20microbiota/Rice%20microbiota-HHRTS/version%20soumise/Revised%20version/Revised%20Version_Last/Supporting%20information/SI_File_S1.html#org1d96c0c)
- [4. FASTQ merging](file:///Users/verniere/Documents/Publications/YYT%20rice%20microbiota/Rice%20microbiota-HHRTS/version%20soumise/Revised%20version/Revised%20Version_Last/Supporting%20information/SI_File_S1.html#org852b415)
  - [4.1. Assembly success rates](file:///Users/verniere/Documents/Publications/YYT%20rice%20microbiota/Rice%20microbiota-HHRTS/version%20soumise/Revised%20version/Revised%20Version_Last/Supporting%20information/SI_File_S1.html#orgfcf5c50)
- [5. 16S reference sequences](file:///Users/verniere/Documents/Publications/YYT%20rice%20microbiota/Rice%20microbiota-HHRTS/version%20soumise/Revised%20version/Revised%20Version_Last/Supporting%20information/SI_File_S1.html#org6c7c322)
- [6. 16S primer clipping (add ee, dereplication and fasta conversion)](file:///Users/verniere/Documents/Publications/YYT%20rice%20microbiota/Rice%20microbiota-HHRTS/version%20soumise/Revised%20version/Revised%20Version_Last/Supporting%20information/SI_File_S1.html#orgffee5dc)
- [7. 16S Global clustering and annotation](file:///Users/verniere/Documents/Publications/YYT%20rice%20microbiota/Rice%20microbiota-HHRTS/version%20soumise/Revised%20version/Revised%20Version_Last/Supporting%20information/SI_File_S1.html#org7766be3)
- [8. 16S OTU table](file:///Users/verniere/Documents/Publications/YYT%20rice%20microbiota/Rice%20microbiota-HHRTS/version%20soumise/Revised%20version/Revised%20Version_Last/Supporting%20information/SI_File_S1.html#org2dcc9bb)
- [9. ITS2 reference sequences](file:///Users/verniere/Documents/Publications/YYT%20rice%20microbiota/Rice%20microbiota-HHRTS/version%20soumise/Revised%20version/Revised%20Version_Last/Supporting%20information/SI_File_S1.html#orga041376)
- [10. ITS2 primer clipping (add ee, dereplication and fasta conversion)](file:///Users/verniere/Documents/Publications/YYT%20rice%20microbiota/Rice%20microbiota-HHRTS/version%20soumise/Revised%20version/Revised%20Version_Last/Supporting%20information/SI_File_S1.html#org7042456)
- [11. ITS2 Global clustering and annotation](file:///Users/verniere/Documents/Publications/YYT%20rice%20microbiota/Rice%20microbiota-HHRTS/version%20soumise/Revised%20version/Revised%20Version_Last/Supporting%20information/SI_File_S1.html#org9f353c2)
- [12. ITS2 OTU table](file:///Users/verniere/Documents/Publications/YYT%20rice%20microbiota/Rice%20microbiota-HHRTS/version%20soumise/Revised%20version/Revised%20Version_Last/Supporting%20information/SI_File_S1.html#orgc39e408)

Pascal Alonso, Laurence Blondin , Pierre Gladieux, Frédéric Mahé, Hervé Sanguin, Romain Ferdinand, Denis Filloux, Eric Desmarais, Frédérique Cerqueira, Baihui Jin, Huichuan Huang, Xiahong He, Jean-Benoit Morel, Darren P. Martin, Philippe Roumagnac and Christian Vernière

supplementary file for bioinformatics methods

**1 Disclaimer**

The purpose of this document is to provide the reader with details on the bioinformatics methods used to prepare this paper. The code snippets and shell commands presented here were executed on a Debian GNU/Linux 10, and might have to be adapted to your particular system. Use them carefully.

**2 Check quality encoding**

# kl

cd ${HOME}/projects/Yunnan_Rice_2016/data/

VSEARCH="${HOME}/bin/vsearch/bin/vsearch"

for FASTQ in ./run_*/*_R1_001.fastq.gz ; do

# Guess phred quality score

ENCODING=$("${VSEARCH}" --fastq_chars "${FASTQ}" 2>&1 | \

grep "^Guess" | grep -o "[0-9][0-9]$")

# Is it a regular encoding?

[[ "${ENCODING}" != 33 ]] && \

[[ "${ENCODING}" != 64 ]] && \

echo "Error: ${f} unknown quality encoding" 1>&2 && break

echo "${FASTQ} ${ENCODING}"

done

All files are in quality offset +33.

**3 R1-R2 quality**

# kl

cd ${HOME}/projects/Yunnan_Rice_2016/data/

VSEARCH="${HOME}/bin/vsearch/bin/vsearch"

for FOLDER in $(ls -1d run_*) ; do

mkdir -p ../results/${FOLDER}

for PAIR in 1 2 ; do

# Summarize read quality

"${VSEARCH}" \

--quiet \

--fastq_eestats <(zcat ./${FOLDER}/*_L001_R${PAIR}_001.fastq.gz) \

--output "../results/${FOLDER}/R${PAIR}_eestats.log"

done

done

# kl

cd ${HOME}/projects/Yunnan_Rice_2016/data/

VSEARCH="${HOME}/bin/vsearch/bin/vsearch"

for FOLDER in $(ls -1d run_16S_250pb_2018062*) ; do

mkdir -p ../results/${FOLDER}

for PAIR in 1 2 ; do

# Summarize read quality

"${VSEARCH}" \

--quiet \

--fastq_eestats <(zcat ./${FOLDER}/*_L001_R${PAIR}_001.fastq.gz) \

--output "../results/${FOLDER}/R${PAIR}_eestats.log"

done

done

**plots**

library(tidyverse)

mylist <- list(c("${HOME}/projects/Yunnan_Rice_2016/results/run_16S_250pb_20180413/",

"Yunnan Rice 2016 16S 341F-785R (MiSeq 2x250 bp, 2018-04-13)"),

c("${HOME}/projects/Yunnan_Rice_2016/results/run_ITS_250pb_20180413/",

"Yunnan Rice 2016 ITS2 (MiSeq 2x250 bp, 2018-04-13)"),

c("${HOME}/projects/Yunnan_Rice_2016/results/run_16S_250pb_20180305/",

"Yunnan Rice 2016 16S 341F-785R (MiSeq 2x250 bp, 2018-03-05)"),

c("${HOME}/projects/Yunnan_Rice_2016/results/run_ITS_250pb_20180307/",

"Yunnan Rice 2016 ITS2 (MiSeq 2x250 bp, 2018-03-07)"),

c("${HOME}/projects/Yunnan_Rice_2016/results/run_250pb/",

"Yunnan Rice 2016 16S 341F-785R (MiSeq 2x250 bp)"))

for (item in mylist) {

output <- "R1_vs_R2_quality.pdf"

setwd(item[[1]])

## Load data

all_sets <- data.frame()

for (read in c("R1", "R2")) {

input <- paste("./", read, "_eestats.log", sep = "")

a <- read.table(input, sep = "\t", header = TRUE) %>%

tbl_df() %>%

select(Pos, Min_EE, Low_EE, Med_EE, Hi_EE, Max_EE) %>%

mutate(read = read)

all_sets <- bind_rows(all_sets, a)

}

## Plot (facets)

ggplot(data = all_sets, aes(x = Pos, y = Med_EE)) +

geom_linerange(aes(ymin = Low_EE, ymax = Hi_EE), colour = "burlywood") +

geom_point(shape = 19, size = 1, color = "firebrick") +

scale_x_continuous() +

theme_bw(base_size = 16) +

ggtitle(item[[2]]) +

xlab("position") +

ylab("median expected error") +

facet_grid(. ~ read) +

coord_cartesian(ylim = c(0, 1.0))

ggsave(output, width = 12, height = 5)

}

quit(save = "no")

**4 FASTQ merging**

# kl

cd ${HOME}/projects/Yunnan_Rice_2016/data/

VSEARCH="${HOME}/bin/vsearch/bin/vsearch"

ENCODING=33

THREADS=4

for FORWARD in ./run_*/*_R1_001.fastq.gz ; do

REVERSE="${FORWARD/_R1_/_R2_}"

OUTPUT="${FORWARD/_L001_R1_001.fastq.gz/}"

"${VSEARCH}" \

--threads ${THREADS} \

--fastq_mergepairs "${FORWARD}" \

--reverse "${REVERSE}" \

--fastq_ascii ${ENCODING} \

--fastqout "${OUTPUT}_assembled.fastq" \

--fastq_allowmergestagger \

--quiet 2> "${OUTPUT}_assembled.log"

done

**4.1 Assembly success rates**

# kl

cd ${HOME}/projects/Yunnan_Rice_2016/data/

for f in ./run_*/*assembled.log ; do

echo -en "${f/.log/}\t"

head -n 3 ${f} | \

awk '{if (NR == 1) {printf "%s\t", $1}

if (NR == 2) {printf "%s\t%s\n", $1, $3}}' | tr -d "()"

done | sed 's/*_assembled/ /g ; s/\.\/// ; s/\//\t/ ; s/_assembled//'

| **run** | **sample** | **available** | **assembled** | **rate** |
| --- | --- | --- | --- | --- |
| run_16S_250pb_20180305 | J2AR_S21 | 73976 | 71040 | 96.0% |
| run_16S_250pb_20180305 | J2AT_S53 | 42232 | 40292 | 95.4% |
| run_16S_250pb_20180305 | SEPYA-A19-2R_S66 | 56935 | 53896 | 94.7% |
| run_16S_250pb_20180305 | SEPYA-A19-2T_S81 | 89614 | 86271 | 96.3% |
| run_16S_250pb_20180305 | SEPYA-A35-2R_S82 | 91906 | 88487 | 96.3% |
| run_16S_250pb_20180305 | SEPYA-A35-2T_S2 | 81618 | 78354 | 96.0% |
| run_16S_250pb_20180305 | SEPYA-B19-1T_S89 | 40524 | 37680 | 93.0% |
| run_16S_250pb_20180305 | SEPYA-B35-2T_S10 | 95456 | 91293 | 95.6% |
| run_16S_250pb_20180305 | SEPYA-R35-2R_S90 | 67513 | 64422 | 95.4% |
| run_16S_250pb_20180305 | T-negatif-extraction2_S296 | 122113 | 118434 | 97.0% |
| run_16S_250pb_20180305 | Tneg-extraction_S3 | 104119 | 100398 | 96.4% |
| run_16S_250pb_20180305 | Tneg-PCR1_S37 | 24302 | 23012 | 94.7% |
| run_16S_250pb_20180305 | Tneg-PCR2_S45 | 17556 | 16905 | 96.3% |
| run_16S_250pb_20180305 | Tpositif-ADN1_S18 | 36662 | 35625 | 97.2% |
| run_16S_250pb_20180305 | T-positif-ADN2_S74 | 27710 | 26912 | 97.1% |
| run_16S_250pb_20180305 | Tpositif-EXRATION_S96 | 94484 | 91301 | 96.6% |
| run_16S_250pb_20180305 | YYM1-bR_S9 | 37605 | 36497 | 97.1% |
| run_16S_250pb_20180305 | YYM1-fR_S41 | 28315 | 27025 | 95.4% |
| run_16S_250pb_20180305 | YYM1-gR_S49 | 54384 | 51962 | 95.5% |
| run_16S_250pb_20180305 | YYM1-hR_S57 | 51572 | 49444 | 95.9% |
| run_16S_250pb_20180305 | YYM1-iR_S65 | 35360 | 34118 | 96.5% |
| run_16S_250pb_20180305 | YYM1-jR_S73 | 42299 | 40037 | 94.7% |
| run_16S_250pb_20180305 | YYM3-fR_S11 | 45840 | 43806 | 95.6% |
| run_16S_250pb_20180305 | YYM3-gR_S19 | 56303 | 53198 | 94.5% |
| run_16S_250pb_20180305 | YYM3-hR_S27 | 34846 | 32760 | 94.0% |
| run_16S_250pb_20180305 | YYM3-iR_S35 | 69311 | 66016 | 95.2% |
| run_16S_250pb_20180305 | YYM3-jR_S43 | 21197 | 19958 | 94.2% |
| run_16S_250pb_20180305 | YYM4-aR_S36 | 88554 | 84704 | 95.7% |
| run_16S_250pb_20180305 | YYM4-bR_S44 | 81296 | 77952 | 95.9% |
| run_16S_250pb_20180305 | YYM4-cR_S52 | 104267 | 100552 | 96.4% |
| run_16S_250pb_20180305 | YYM4-dR_S60 | 112503 | 109364 | 97.2% |
| run_16S_250pb_20180305 | YYM4-eR_S68 | 129659 | 124849 | 96.3% |
| run_16S_250pb_20180305 | YYM4-fR_S76 | 46216 | 44094 | 95.4% |
| run_16S_250pb_20180305 | YYM4-gR_S84 | 33865 | 33123 | 97.8% |
| run_16S_250pb_20180305 | YYM4-hR_S92 | 58127 | 54712 | 94.1% |
| run_16S_250pb_20180305 | YYM4-iR_S5 | 21636 | 20809 | 96.2% |
| run_16S_250pb_20180305 | YYM4-jR_S13 | 41555 | 40008 | 96.3% |
| run_16S_250pb_20180305 | YYM5-aR_S6 | 33497 | 32105 | 95.8% |
| run_16S_250pb_20180305 | YYM5-bR_S14 | 63111 | 60237 | 95.4% |
| run_16S_250pb_20180305 | YYM5-cR_S22 | 84518 | 80280 | 95.0% |
| run_16S_250pb_20180305 | YYM5-dR_S30 | 51559 | 49786 | 96.6% |
| run_16S_250pb_20180305 | YYM5-eR_S38 | 46637 | 44051 | 94.5% |
| run_16S_250pb_20180305 | YYM5-fR_S46 | 58518 | 56573 | 96.7% |
| run_16S_250pb_20180305 | YYM5-gR_S54 | 47610 | 45272 | 95.1% |
| run_16S_250pb_20180305 | YYM5-hR_S62 | 35630 | 33782 | 94.8% |
| run_16S_250pb_20180305 | YYM5-iR_S70 | 55334 | 53759 | 97.2% |
| run_16S_250pb_20180305 | YYM5-jR_S78 | 50196 | 48215 | 96.1% |
| run_16S_250pb_20180305 | YYM6-aR_S71 | 38832 | 36660 | 94.4% |
| run_16S_250pb_20180305 | YYM6-bR_S79 | 60713 | 58368 | 96.1% |
| run_16S_250pb_20180305 | YYM6-cR_S87 | 59974 | 57900 | 96.5% |
| run_16S_250pb_20180305 | YYM6-dR_S95 | 85521 | 81249 | 95.0% |
| run_16S_250pb_20180305 | YYM6-eR_S8 | 37901 | 36490 | 96.3% |
| run_16S_250pb_20180305 | YYM6-fR_S16 | 83805 | 80746 | 96.3% |
| run_16S_250pb_20180305 | YYM6-gR_S24 | 59577 | 57322 | 96.2% |
| run_16S_250pb_20180305 | YYM6-hR_S32 | 35002 | 33124 | 94.6% |
| run_16S_250pb_20180305 | YYM6-iR_S40 | 33324 | 31325 | 94.0% |
| run_16S_250pb_20180305 | YYM6-jR_S48 | 30666 | 29430 | 96.0% |
| run_16S_250pb_20180305 | YYM7-aR_S241 | 63264 | 60553 | 95.7% |
| run_16S_250pb_20180305 | YYM7-cR_S257 | 84135 | 80067 | 95.2% |
| run_16S_250pb_20180305 | YYM7-dR_S265 | 50147 | 47249 | 94.2% |
| run_16S_250pb_20180305 | YYM7-eR_S273 | 57607 | 54659 | 94.9% |
| run_16S_250pb_20180305 | YYM7-fR_S281 | 51254 | 47836 | 93.3% |
| run_16S_250pb_20180305 | YYM7-gR_S289 | 45144 | 43251 | 95.8% |
| run_16S_250pb_20180305 | YYM7-hR_S202 | 47206 | 43960 | 93.1% |
| run_16S_250pb_20180305 | YYM7-iR_S210 | 26925 | 25388 | 94.3% |
| run_16S_250pb_20180305 | YYM7-jR_S218 | 35263 | 33000 | 93.6% |
| run_16S_250pb_20180305 | YYM8-aR_S211 | 40525 | 38750 | 95.6% |
| run_16S_250pb_20180305 | YYM8-bR_S219 | 39974 | 38437 | 96.2% |
| run_16S_250pb_20180305 | YYM8-cR_S227 | 51259 | 48855 | 95.3% |
| run_16S_250pb_20180305 | YYM8-dR_S235 | 49599 | 48134 | 97.0% |
| run_16S_250pb_20180305 | YYM8-eR_S243 | 42607 | 40573 | 95.2% |
| run_16S_250pb_20180305 | YYM8-fR_S251 | 79949 | 76490 | 95.7% |
| run_16S_250pb_20180305 | YYM8-gR_S259 | 48773 | 45897 | 94.1% |
| run_16S_250pb_20180305 | YYM8-hR_S267 | 58468 | 55945 | 95.7% |
| run_16S_250pb_20180305 | YYM8-iR_S275 | 57497 | 54664 | 95.1% |
| run_16S_250pb_20180305 | YYM8-jR_S283 | 31823 | 30238 | 95.0% |
| run_16S_250pb_20180305 | YYM9-aR_S276 | 92221 | 88589 | 96.1% |
| run_16S_250pb_20180305 | YYM9-bR_S284 | 56693 | 53341 | 94.1% |
| run_16S_250pb_20180305 | YYM9-cR_S292 | 67227 | 63672 | 94.7% |
| run_16S_250pb_20180305 | YYM9-dR_S205 | 43894 | 41684 | 95.0% |
| run_16S_250pb_20180305 | YYM9-eR_S213 | 83822 | 80071 | 95.5% |
| run_16S_250pb_20180305 | YYM9-fR_S221 | 90752 | 85055 | 93.7% |
| run_16S_250pb_20180305 | YYM9-gR_S229 | 66552 | 64152 | 96.4% |
| run_16S_250pb_20180305 | YYM9-hR_S237 | 57127 | 53973 | 94.5% |
| run_16S_250pb_20180305 | YYM9-iR_S245 | 48119 | 43794 | 91.0% |
| run_16S_250pb_20180305 | YYM9-jR_S253 | 66086 | 62512 | 94.6% |
| run_16S_250pb_20180305 | YYR5-jR_S51 | 55568 | 53318 | 96.0% |
| run_16S_250pb_20180305 | YYR7-aR_S291 | 41536 | 39262 | 94.5% |
| run_16S_250pb_20180305 | YYT10-aR_S231 | 42577 | 39724 | 93.3% |
| run_16S_250pb_20180305 | YYT10-bR_S239 | 80248 | 77464 | 96.5% |
| run_16S_250pb_20180305 | YYT10-cR_S247 | 39720 | 37789 | 95.1% |
| run_16S_250pb_20180305 | YYT10-dR_S255 | 44817 | 42258 | 94.3% |
| run_16S_250pb_20180305 | YYT10-eR_S263 | 89956 | 87349 | 97.1% |
| run_16S_250pb_20180305 | YYT10-fR_S271 | 81578 | 77894 | 95.5% |
| run_16S_250pb_20180305 | YYT10-gR_S279 | 28798 | 26771 | 93.0% |
| run_16S_250pb_20180305 | YYT10-hR_S287 | 50114 | 48423 | 96.6% |
| run_16S_250pb_20180305 | YYT10-iR_S295 | 75157 | 70957 | 94.4% |
| run_16S_250pb_20180305 | YYT10-jR_S208 | 83587 | 81156 | 97.1% |
| run_16S_250pb_20180305 | YYT11-aR_S216 | 70220 | 66470 | 94.7% |
| run_16S_250pb_20180305 | YYT11-bR_S224 | 58327 | 55139 | 94.5% |
| run_16S_250pb_20180305 | YYT11-cR_S232 | 37014 | 35162 | 95.0% |
| run_16S_250pb_20180305 | YYT11-dR_S240 | 61110 | 58264 | 95.3% |
| run_16S_250pb_20180305 | YYT11-eR_S248 | 50149 | 47233 | 94.2% |
| run_16S_250pb_20180305 | YYT11-fR_S256 | 71272 | 67533 | 94.8% |
| run_16S_250pb_20180305 | YYT11-gR_S264 | 87356 | 83085 | 95.1% |
| run_16S_250pb_20180305 | YYT11-hR_S272 | 71972 | 67657 | 94.0% |
| run_16S_250pb_20180305 | YYT11-iR_S280 | 60661 | 58281 | 96.1% |
| run_16S_250pb_20180305 | YYT11-jR_S288 | 75539 | 71746 | 95.0% |
| run_16S_250pb_20180305 | YYT1-fR_S26 | 52624 | 50624 | 96.2% |
| run_16S_250pb_20180305 | YYT1-gR_S34 | 39817 | 37362 | 93.8% |
| run_16S_250pb_20180305 | YYT1-hR_S42 | 46449 | 44401 | 95.6% |
| run_16S_250pb_20180305 | YYT1-iR_S50 | 39834 | 37511 | 94.2% |
| run_16S_250pb_20180305 | YYT1-jR_S58 | 69875 | 65909 | 94.3% |
| run_16S_250pb_20180305 | YYT2-cR_S67 | 21901 | 20657 | 94.3% |
| run_16S_250pb_20180305 | YYT2-fR_S91 | 38385 | 35882 | 93.5% |
| run_16S_250pb_20180305 | YYT2-gR_S4 | 44718 | 42515 | 95.1% |
| run_16S_250pb_20180305 | YYT2-hR_S12 | 55418 | 52033 | 93.9% |
| run_16S_250pb_20180305 | YYT2-iR_S20 | 41590 | 39106 | 94.0% |
| run_16S_250pb_20180305 | YYT2-jR_S28 | 55740 | 52818 | 94.8% |
| run_16S_250pb_20180305 | YYT3-bR_S29 | 38261 | 35425 | 92.6% |
| run_16S_250pb_20180305 | YYT3-fR_S61 | 19839 | 18710 | 94.3% |
| run_16S_250pb_20180305 | YYT3-gR_S69 | 14956 | 14005 | 93.6% |
| run_16S_250pb_20180305 | YYT3-hR_S77 | 33038 | 31529 | 95.4% |
| run_16S_250pb_20180305 | YYT3-iR_S85 | 58253 | 54555 | 93.7% |
| run_16S_250pb_20180305 | YYT3-jR_S93 | 71336 | 67377 | 94.5% |
| run_16S_250pb_20180305 | YYT4-aR_S86 | 78069 | 74192 | 95.0% |
| run_16S_250pb_20180305 | YYT4-bR_S94 | 72493 | 68310 | 94.2% |
| run_16S_250pb_20180305 | YYT4-cR_S7 | 50474 | 48443 | 96.0% |
| run_16S_250pb_20180305 | YYT4-dR_S15 | 39043 | 36993 | 94.7% |
| run_16S_250pb_20180305 | YYT4-eR_S23 | 42341 | 40450 | 95.5% |
| run_16S_250pb_20180305 | YYT4-fR_S31 | 56433 | 53483 | 94.8% |
| run_16S_250pb_20180305 | YYT4-gR_S39 | 55054 | 52262 | 94.9% |
| run_16S_250pb_20180305 | YYT4-hR_S47 | 44241 | 42160 | 95.3% |
| run_16S_250pb_20180305 | YYT4-iR_S55 | 65424 | 62096 | 94.9% |
| run_16S_250pb_20180305 | YYT4-jR_S63 | 59015 | 56283 | 95.4% |
| run_16S_250pb_20180305 | YYT5-aR_S56 | 39002 | 37160 | 95.3% |
| run_16S_250pb_20180305 | YYT5-bR_S249 | 70956 | 68170 | 96.1% |
| run_16S_250pb_20180305 | YYT5-bR_S64 | 65056 | 62439 | 96.0% |
| run_16S_250pb_20180305 | YYT5-cR_S72 | 44600 | 41855 | 93.8% |
| run_16S_250pb_20180305 | YYT5-dR_S80 | 40711 | 38934 | 95.6% |
| run_16S_250pb_20180305 | YYT5-eR_S88 | 74020 | 70657 | 95.5% |
| run_16S_250pb_20180305 | YYT5-fR_S1 | 97702 | 94319 | 96.5% |
| run_16S_250pb_20180305 | YYT5-fR_S201 | 104586 | 100110 | 95.7% |
| run_16S_250pb_20180305 | YYT5-fT_S59 | 68383 | 63639 | 93.1% |
| run_16S_250pb_20180305 | YYT5-gR_S17 | 37780 | 35750 | 94.6% |
| run_16S_250pb_20180305 | YYT5-gR_S209 | 51194 | 48310 | 94.4% |
| run_16S_250pb_20180305 | YYT5-gT_S75 | 94142 | 90706 | 96.4% |
| run_16S_250pb_20180305 | YYT5-hR_S217 | 86376 | 83286 | 96.4% |
| run_16S_250pb_20180305 | YYT5-hR_S25 | 54640 | 51313 | 93.9% |
| run_16S_250pb_20180305 | YYT5-hT_S83 | 76938 | 73123 | 95.0% |
| run_16S_250pb_20180305 | YYT5-iR_S225 | 92406 | 88303 | 95.6% |
| run_16S_250pb_20180305 | YYT5-iR_S33 | 42583 | 40596 | 95.3% |
| run_16S_250pb_20180305 | YYT5-jR_S233 | 54124 | 50794 | 93.8% |
| run_16S_250pb_20180305 | YYT6-aR_S226 | 18704 | 17731 | 94.8% |
| run_16S_250pb_20180305 | YYT6-bR_S234 | 51572 | 50689 | 98.3% |
| run_16S_250pb_20180305 | YYT6-cR_S242 | 57741 | 55678 | 96.4% |
| run_16S_250pb_20180305 | YYT6-dR_S250 | 42527 | 40410 | 95.0% |
| run_16S_250pb_20180305 | YYT6-eR_S258 | 58702 | 56263 | 95.8% |
| run_16S_250pb_20180305 | YYT6-fR_S266 | 46384 | 44436 | 95.8% |
| run_16S_250pb_20180305 | YYT6-gR_S274 | 54374 | 51635 | 95.0% |
| run_16S_250pb_20180305 | YYT6-hR_S282 | 45759 | 43283 | 94.6% |
| run_16S_250pb_20180305 | YYT6-iR_S290 | 56120 | 53245 | 94.9% |
| run_16S_250pb_20180305 | YYT6-jR_S203 | 47074 | 44397 | 94.3% |
| run_16S_250pb_20180305 | YYT7-bR_S204 | 48770 | 46155 | 94.6% |
| run_16S_250pb_20180305 | YYT7-cR_S212 | 78320 | 75214 | 96.0% |
| run_16S_250pb_20180305 | YYT7-dR_S220 | 96090 | 92336 | 96.1% |
| run_16S_250pb_20180305 | YYT7-eR_S228 | 43395 | 40737 | 93.9% |
| run_16S_250pb_20180305 | YYT7-fR_S236 | 31202 | 29393 | 94.2% |
| run_16S_250pb_20180305 | YYT7-gR_S244 | 94311 | 91237 | 96.7% |
| run_16S_250pb_20180305 | YYT7-hR_S252 | 48319 | 46666 | 96.6% |
| run_16S_250pb_20180305 | YYT7-iR_S260 | 79934 | 76297 | 95.4% |
| run_16S_250pb_20180305 | YYT7-jR_S268 | 86947 | 83163 | 95.6% |
| run_16S_250pb_20180305 | YYT8-aR_S261 | 77191 | 74216 | 96.1% |
| run_16S_250pb_20180305 | YYT8-bR_S269 | 80268 | 77443 | 96.5% |
| run_16S_250pb_20180305 | YYT8-cR_S277 | 67229 | 64537 | 96.0% |
| run_16S_250pb_20180305 | YYT8-dR_S285 | 91810 | 90355 | 98.4% |
| run_16S_250pb_20180305 | YYT8-eR_S293 | 59406 | 56310 | 94.8% |
| run_16S_250pb_20180305 | YYT8-fR_S206 | 80524 | 78450 | 97.4% |
| run_16S_250pb_20180305 | YYT8-gR_S214 | 79543 | 75960 | 95.5% |
| run_16S_250pb_20180305 | YYT8-hR_S222 | 81604 | 79204 | 97.1% |
| run_16S_250pb_20180305 | YYT8-iR_S230 | 48087 | 45427 | 94.5% |
| run_16S_250pb_20180305 | YYT8-jR_S238 | 70204 | 68014 | 96.9% |
| run_16S_250pb_20180305 | YYT9-aR_S246 | 46451 | 44371 | 95.5% |
| run_16S_250pb_20180305 | YYT9-bR_S254 | 45427 | 42910 | 94.5% |
| run_16S_250pb_20180305 | YYT9-cR_S262 | 98359 | 95253 | 96.8% |
| run_16S_250pb_20180305 | YYT9-dR_S270 | 40817 | 38767 | 95.0% |
| run_16S_250pb_20180305 | YYT9-eR_S278 | 47797 | 45887 | 96.0% |
| run_16S_250pb_20180305 | YYT9-fR_S286 | 19350 | 18466 | 95.4% |
| run_16S_250pb_20180305 | YYT9-gR_S294 | 49007 | 46586 | 95.1% |
| run_16S_250pb_20180305 | YYT9-hR_S207 | 65033 | 62138 | 95.5% |
| run_16S_250pb_20180305 | YYT9-iR_S215 | 61827 | 58858 | 95.2% |
| run_16S_250pb_20180305 | YYT9-jR_S223 | 87244 | 82240 | 94.3% |
| run_ITS_250pb_20180307 | J2AT_S149 | 1 | 1 | 100.0% |
| run_ITS_250pb_20180307 | SEPYA-A19-2R_S162 | 65330 | 39206 | 60.0% |
| run_ITS_250pb_20180307 | SEPYA-A19-2T_S177 | 43614 | 33295 | 76.3% |
| run_ITS_250pb_20180307 | SEPYA-A35-2R_S178 | 44855 | 29510 | 65.8% |
| run_ITS_250pb_20180307 | SEPYA-A35-2T_S98 | 47315 | 33740 | 71.3% |
| run_ITS_250pb_20180307 | SEPYA-B19-1T_S185 | 49453 | 32578 | 65.9% |
| run_ITS_250pb_20180307 | SEPYA-B35-2T_S106 | 15352 | 8022 | 52.3% |
| run_ITS_250pb_20180307 | SEPYA-B57-2R_S170 | 42758 | 29451 | 68.9% |
| run_ITS_250pb_20180307 | SEPYA-R35-2R_S186 | 54519 | 29846 | 54.7% |
| run_ITS_250pb_20180307 | T-Negatif-extraction_S384 | 21884 | 15862 | 72.5% |
| run_ITS_250pb_20180307 | T-negatif-extraction_S99 | 36 | 13 | 36.1% |
| run_ITS_250pb_20180307 | T-Negatif-PCR1_S133 | 6 | 2 | 33.3% |
| run_ITS_250pb_20180307 | T-Negatif-PCR2_S141 | 6 | 1 | 16.7% |
| run_ITS_250pb_20180307 | T-positif-ADN1_S114 | 26 | 0 | 0.0% |
| run_ITS_250pb_20180307 | T-positif-ADN2_S117 | 1 | 0 | 0.0% |
| run_ITS_250pb_20180307 | T-POSITIF-EXRATION_S192 | 126367 | 90016 | 71.2% |
| run_ITS_250pb_20180307 | YYM1-fR_S137 | 36250 | 17008 | 46.9% |
| run_ITS_250pb_20180307 | YYM1-gR_S145 | 22802 | 10124 | 44.4% |
| run_ITS_250pb_20180307 | YYM1-hR_S153 | 45943 | 29675 | 64.6% |
| run_ITS_250pb_20180307 | YYM1-iR_S161 | 52493 | 23116 | 44.0% |
| run_ITS_250pb_20180307 | YYM1-jR_S169 | 54700 | 37400 | 68.4% |
| run_ITS_250pb_20180307 | YYM3-fR_S107 | 33333 | 16096 | 48.3% |
| run_ITS_250pb_20180307 | YYM3-gR_S115 | 34611 | 23434 | 67.7% |
| run_ITS_250pb_20180307 | YYM3-hR_S123 | 20014 | 7119 | 35.6% |
| run_ITS_250pb_20180307 | YYM3-iR_S131 | 28653 | 15849 | 55.3% |
| run_ITS_250pb_20180307 | YYM3-jR_S139 | 34877 | 13440 | 38.5% |
| run_ITS_250pb_20180307 | YYM4-aR_S132 | 78678 | 51480 | 65.4% |
| run_ITS_250pb_20180307 | YYM4-bR_S140 | 70252 | 52826 | 75.2% |
| run_ITS_250pb_20180307 | YYM4-cR_S148 | 36025 | 29864 | 82.9% |
| run_ITS_250pb_20180307 | YYM4-dR_S156 | 2623 | 2014 | 76.8% |
| run_ITS_250pb_20180307 | YYM4-eR_S164 | 9 | 1 | 11.1% |
| run_ITS_250pb_20180307 | YYM4-fR_S172 | 53303 | 29330 | 55.0% |
| run_ITS_250pb_20180307 | YYM4-gR_S180 | 33218 | 20042 | 60.3% |
| run_ITS_250pb_20180307 | YYM4-hR_S188 | 61543 | 13176 | 21.4% |
| run_ITS_250pb_20180307 | YYM4-iR_S101 | 23490 | 10581 | 45.0% |
| run_ITS_250pb_20180307 | YYM4-jR_S109 | 48335 | 27985 | 57.9% |
| run_ITS_250pb_20180307 | YYM5-aR_S102 | 45491 | 27325 | 60.1% |
| run_ITS_250pb_20180307 | YYM5-bR_S110 | 22062 | 10710 | 48.5% |
| run_ITS_250pb_20180307 | YYM5-cR_S118 | 61530 | 43152 | 70.1% |
| run_ITS_250pb_20180307 | YYM5-dR_S126 | 42360 | 30525 | 72.1% |
| run_ITS_250pb_20180307 | YYM5-eR_S134 | 1473 | 950 | 64.5% |
| run_ITS_250pb_20180307 | YYM5-fR_S142 | 32948 | 4561 | 13.8% |
| run_ITS_250pb_20180307 | YYM5-gR_S150 | 76233 | 55538 | 72.9% |
| run_ITS_250pb_20180307 | YYM5-hR_S158 | 109222 | 87520 | 80.1% |
| run_ITS_250pb_20180307 | YYM5-iR_S166 | 13384 | 5897 | 44.1% |
| run_ITS_250pb_20180307 | YYM5-jR_S174 | 29888 | 15648 | 52.4% |
| run_ITS_250pb_20180307 | YYM6-aR_S167 | 25230 | 10386 | 41.2% |
| run_ITS_250pb_20180307 | YYM6-bR_S175 | 10565 | 4545 | 43.0% |
| run_ITS_250pb_20180307 | YYM6-cR_S183 | 40482 | 25002 | 61.8% |
| run_ITS_250pb_20180307 | YYM6-dR_S191 | 44336 | 27707 | 62.5% |
| run_ITS_250pb_20180307 | YYM6-eR_S104 | 78129 | 46842 | 60.0% |
| run_ITS_250pb_20180307 | YYM6-fR_S112 | 30746 | 16936 | 55.1% |
| run_ITS_250pb_20180307 | YYM6-gR_S120 | 97382 | 75794 | 77.8% |
| run_ITS_250pb_20180307 | YYM6-hR_S128 | 58850 | 42355 | 72.0% |
| run_ITS_250pb_20180307 | YYM6-iR_S136 | 60437 | 32623 | 54.0% |
| run_ITS_250pb_20180307 | YYM6-jR_S144 | 69590 | 31254 | 44.9% |
| run_ITS_250pb_20180307 | YYM7-aR_S329 | 54839 | 34252 | 62.5% |
| run_ITS_250pb_20180307 | YYM7-cR_S345 | 50955 | 28015 | 55.0% |
| run_ITS_250pb_20180307 | YYM7-dR_S353 | 29498 | 11072 | 37.5% |
| run_ITS_250pb_20180307 | YYM7-eR_S361 | 63704 | 22259 | 34.9% |
| run_ITS_250pb_20180307 | YYM7-fR_S369 | 16803 | 6295 | 37.5% |
| run_ITS_250pb_20180307 | YYM7-gR_S377 | 22348 | 13081 | 58.5% |
| run_ITS_250pb_20180307 | YYM7-hR_S290 | 17635 | 6137 | 34.8% |
| run_ITS_250pb_20180307 | YYM7-iR_S298 | 17207 | 7495 | 43.6% |
| run_ITS_250pb_20180307 | YYM7-jR_S306 | 29068 | 14165 | 48.7% |
| run_ITS_250pb_20180307 | YYM8-aR_S299 | 22325 | 10399 | 46.6% |
| run_ITS_250pb_20180307 | YYM8-bR_S307 | 35492 | 20897 | 58.9% |
| run_ITS_250pb_20180307 | YYM8-cR_S315 | 33302 | 19276 | 57.9% |
| run_ITS_250pb_20180307 | YYM8-dR_S323 | 39324 | 26469 | 67.3% |
| run_ITS_250pb_20180307 | YYM8-eR_S331 | 20919 | 3356 | 16.0% |
| run_ITS_250pb_20180307 | YYM8-fR_S339 | 36885 | 20155 | 54.6% |
| run_ITS_250pb_20180307 | YYM8-gR_S347 | 31976 | 17753 | 55.5% |
| run_ITS_250pb_20180307 | YYM8-hR_S355 | 39824 | 22351 | 56.1% |
| run_ITS_250pb_20180307 | YYM8-iR_S363 | 23130 | 13714 | 59.3% |
| run_ITS_250pb_20180307 | YYM8-jR_S371 | 32721 | 17322 | 52.9% |
| run_ITS_250pb_20180307 | YYM9-aR_S364 | 76947 | 13917 | 18.1% |
| run_ITS_250pb_20180307 | YYM9-bR_S372 | 22851 | 11310 | 49.5% |
| run_ITS_250pb_20180307 | YYM9-cR_S380 | 35348 | 18845 | 53.3% |
| run_ITS_250pb_20180307 | YYM9-dR_S293 | 22180 | 7880 | 35.5% |
| run_ITS_250pb_20180307 | YYM9-eR_S301 | 41745 | 22849 | 54.7% |
| run_ITS_250pb_20180307 | YYM9-fR_S309 | 20662 | 4603 | 22.3% |
| run_ITS_250pb_20180307 | YYM9-gR_S317 | 43667 | 25515 | 58.4% |
| run_ITS_250pb_20180307 | YYM9-hR_S325 | 47099 | 20319 | 43.1% |
| run_ITS_250pb_20180307 | YYM9-iR_S333 | 17393 | 6287 | 36.1% |
| run_ITS_250pb_20180307 | YYM9-jR_S341 | 27057 | 9749 | 36.0% |
| run_ITS_250pb_20180307 | YYT10-aR_S319 | 13314 | 4276 | 32.1% |
| run_ITS_250pb_20180307 | YYT10-bR_S327 | 34294 | 20837 | 60.8% |
| run_ITS_250pb_20180307 | YYT10-cR_S335 | 18371 | 8091 | 44.0% |
| run_ITS_250pb_20180307 | YYT10-dR_S343 | 41719 | 25240 | 60.5% |
| run_ITS_250pb_20180307 | YYT10-eR_S351 | 53433 | 32512 | 60.8% |
| run_ITS_250pb_20180307 | YYT10-fR_S359 | 49017 | 28953 | 59.1% |
| run_ITS_250pb_20180307 | YYT10-gR_S367 | 25181 | 3241 | 12.9% |
| run_ITS_250pb_20180307 | YYT10-hR_S375 | 62230 | 43490 | 69.9% |
| run_ITS_250pb_20180307 | YYT10-iR_S383 | 21779 | 11425 | 52.5% |
| run_ITS_250pb_20180307 | YYT10-jR_S296 | 59112 | 28990 | 49.0% |
| run_ITS_250pb_20180307 | YYT11-aR_S304 | 53753 | 25090 | 46.7% |
| run_ITS_250pb_20180307 | YYT11-bR_S312 | 42518 | 22750 | 53.5% |
| run_ITS_250pb_20180307 | YYT11-cR_S320 | 27284 | 13751 | 50.4% |
| run_ITS_250pb_20180307 | YYT11-dR_S328 | 61092 | 39805 | 65.2% |
| run_ITS_250pb_20180307 | YYT11-eR_S336 | 17717 | 8344 | 47.1% |
| run_ITS_250pb_20180307 | YYT11-fR_S344 | 44960 | 18646 | 41.5% |
| run_ITS_250pb_20180307 | YYT11-gR_S352 | 41918 | 11967 | 28.5% |
| run_ITS_250pb_20180307 | YYT11-hR_S360 | 30134 | 9149 | 30.4% |
| run_ITS_250pb_20180307 | YYT11-iR_S368 | 65633 | 40976 | 62.4% |
| run_ITS_250pb_20180307 | YYT11-jR_S376 | 76655 | 52612 | 68.6% |
| run_ITS_250pb_20180307 | YYT1-bR_S105 | 29737 | 17699 | 59.5% |
| run_ITS_250pb_20180307 | YYT1-fR_S122 | 52896 | 22737 | 43.0% |
| run_ITS_250pb_20180307 | YYT1-gR_S130 | 17587 | 6032 | 34.3% |
| run_ITS_250pb_20180307 | YYT1-hR_S138 | 58617 | 34586 | 59.0% |
| run_ITS_250pb_20180307 | YYT1-iR_S146 | 36639 | 18082 | 49.4% |
| run_ITS_250pb_20180307 | YYT1-jR_S154 | 73018 | 11860 | 16.2% |
| run_ITS_250pb_20180307 | YYT2-cR_S163 | 84297 | 39603 | 47.0% |
| run_ITS_250pb_20180307 | YYT2-fR_S187 | 93041 | 26080 | 28.0% |
| run_ITS_250pb_20180307 | YYT2-gR_S100 | 9432 | 2303 | 24.4% |
| run_ITS_250pb_20180307 | YYT2-hR_S108 | 40386 | 17147 | 42.5% |
| run_ITS_250pb_20180307 | YYT2-iR_S116 | 41487 | 15486 | 37.3% |
| run_ITS_250pb_20180307 | YYT2-jR_S124 | 18938 | 4788 | 25.3% |
| run_ITS_250pb_20180307 | YYT3-bR_S125 | 12614 | 1438 | 11.4% |
| run_ITS_250pb_20180307 | YYT3-fR_S157 | 41149 | 14775 | 35.9% |
| run_ITS_250pb_20180307 | YYT3-gR_S165 | 65132 | 44510 | 68.3% |
| run_ITS_250pb_20180307 | YYT3-hR_S173 | 52358 | 15535 | 29.7% |
| run_ITS_250pb_20180307 | YYT3-iR_S181 | 65244 | 31217 | 47.8% |
| run_ITS_250pb_20180307 | YYT3-jR_S189 | 43031 | 3943 | 9.2% |
| run_ITS_250pb_20180307 | YYT4-aR_S182 | 33502 | 14369 | 42.9% |
| run_ITS_250pb_20180307 | YYT4-bR_S190 | 43625 | 3583 | 8.2% |
| run_ITS_250pb_20180307 | YYT4-cR_S103 | 37953 | 17507 | 46.1% |
| run_ITS_250pb_20180307 | YYT4-dR_S111 | 14752 | 4143 | 28.1% |
| run_ITS_250pb_20180307 | YYT4-eR_S119 | 40474 | 15802 | 39.0% |
| run_ITS_250pb_20180307 | YYT4-fR_S127 | 45406 | 30177 | 66.5% |
| run_ITS_250pb_20180307 | YYT4-gR_S135 | 589 | 53 | 9.0% |
| run_ITS_250pb_20180307 | YYT4-hR_S143 | 36702 | 7083 | 19.3% |
| run_ITS_250pb_20180307 | YYT4-iR_S151 | 71275 | 31267 | 43.9% |
| run_ITS_250pb_20180307 | YYT4-jR_S159 | 76038 | 27279 | 35.9% |
| run_ITS_250pb_20180307 | YYT5-aR_S152 | 48378 | 26899 | 55.6% |
| run_ITS_250pb_20180307 | YYT5-bR_S160 | 19527 | 4939 | 25.3% |
| run_ITS_250pb_20180307 | YYT5-bR_S337 | 67203 | 36200 | 53.9% |
| run_ITS_250pb_20180307 | YYT5-cR_S168 | 41094 | 9071 | 22.1% |
| run_ITS_250pb_20180307 | YYT5-dR_S176 | 52465 | 12695 | 24.2% |
| run_ITS_250pb_20180307 | YYT5-eR_S184 | 31568 | 13395 | 42.4% |
| run_ITS_250pb_20180307 | YYT5-fR_S289 | 44407 | 25698 | 57.9% |
| run_ITS_250pb_20180307 | YYT5-fR_S97 | 26919 | 13183 | 49.0% |
| run_ITS_250pb_20180307 | YYT5-fT_S155 | 89322 | 60262 | 67.5% |
| run_ITS_250pb_20180307 | YYT5-gR_S113 | 27081 | 15651 | 57.8% |
| run_ITS_250pb_20180307 | YYT5-gR_S297 | 22786 | 9641 | 42.3% |
| run_ITS_250pb_20180307 | YYT5-gT_S171 | 25186 | 15282 | 60.7% |
| run_ITS_250pb_20180307 | YYT5-hR_S121 | 34356 | 13365 | 38.9% |
| run_ITS_250pb_20180307 | YYT5-hR_S305 | 48067 | 33717 | 70.1% |
| run_ITS_250pb_20180307 | YYT5-hT_S179 | 33508 | 22205 | 66.3% |
| run_ITS_250pb_20180307 | YYT5-iR_S129 | 23883 | 7380 | 30.9% |
| run_ITS_250pb_20180307 | YYT5-iR_S313 | 67474 | 44481 | 65.9% |
| run_ITS_250pb_20180307 | YYT5-jR_S147 | 44429 | 30868 | 69.5% |
| run_ITS_250pb_20180307 | YYT5-jR_S321 | 42781 | 18674 | 43.7% |
| run_ITS_250pb_20180307 | YYT6-aR_S314 | 14533 | 7335 | 50.5% |
| run_ITS_250pb_20180307 | YYT6-bR_S322 | 76368 | 59288 | 77.6% |
| run_ITS_250pb_20180307 | YYT6-cR_S330 | 31020 | 16826 | 54.2% |
| run_ITS_250pb_20180307 | YYT6-dR_S338 | 32628 | 18665 | 57.2% |
| run_ITS_250pb_20180307 | YYT6-eR_S346 | 96665 | 65196 | 67.4% |
| run_ITS_250pb_20180307 | YYT6-fR_S354 | 32655 | 16539 | 50.6% |
| run_ITS_250pb_20180307 | YYT6-gR_S362 | 15989 | 8498 | 53.1% |
| run_ITS_250pb_20180307 | YYT6-hR_S370 | 34832 | 14184 | 40.7% |
| run_ITS_250pb_20180307 | YYT6-iR_S378 | 45337 | 23631 | 52.1% |
| run_ITS_250pb_20180307 | YYT6-jR_S291 | 25399 | 12577 | 49.5% |
| run_ITS_250pb_20180307 | YYT7-aR_S379 | 17561 | 7226 | 41.1% |
| run_ITS_250pb_20180307 | YYT7-bR_S292 | 41518 | 21021 | 50.6% |
| run_ITS_250pb_20180307 | YYT7-cR_S300 | 50375 | 26857 | 53.3% |
| run_ITS_250pb_20180307 | YYT7-dR_S308 | 40021 | 27872 | 69.6% |
| run_ITS_250pb_20180307 | YYT7-eR_S316 | 27790 | 15627 | 56.2% |
| run_ITS_250pb_20180307 | YYT7-fR_S324 | 30946 | 17070 | 55.2% |
| run_ITS_250pb_20180307 | YYT7-gR_S332 | 81223 | 52005 | 64.0% |
| run_ITS_250pb_20180307 | YYT7-hR_S340 | 94393 | 74409 | 78.8% |
| run_ITS_250pb_20180307 | YYT7-iR_S348 | 43762 | 25033 | 57.2% |
| run_ITS_250pb_20180307 | YYT7-jR_S356 | 64052 | 46080 | 71.9% |
| run_ITS_250pb_20180307 | YYT8-aR_S349 | 40524 | 23702 | 58.5% |
| run_ITS_250pb_20180307 | YYT8-bR_S357 | 83845 | 36111 | 43.1% |
| run_ITS_250pb_20180307 | YYT8-cR_S365 | 61412 | 21665 | 35.3% |
| run_ITS_250pb_20180307 | YYT8-dR_S373 | 74607 | 54464 | 73.0% |
| run_ITS_250pb_20180307 | YYT8-eR_S381 | 19829 | 8331 | 42.0% |
| run_ITS_250pb_20180307 | YYT8-fR_S294 | 53369 | 30330 | 56.8% |
| run_ITS_250pb_20180307 | YYT8-gR_S302 | 63901 | 32497 | 50.9% |
| run_ITS_250pb_20180307 | YYT8-hR_S310 | 47109 | 32295 | 68.6% |
| run_ITS_250pb_20180307 | YYT8-iR_S318 | 23711 | 10724 | 45.2% |
| run_ITS_250pb_20180307 | YYT8-jR_S326 | 51392 | 29499 | 57.4% |
| run_ITS_250pb_20180307 | YYT9-aR_S334 | 66846 | 28765 | 43.0% |
| run_ITS_250pb_20180307 | YYT9-bR_S342 | 22969 | 12330 | 53.7% |
| run_ITS_250pb_20180307 | YYT9-cR_S350 | 49084 | 30502 | 62.1% |
| run_ITS_250pb_20180307 | YYT9-dR_S358 | 35568 | 19960 | 56.1% |
| run_ITS_250pb_20180307 | YYT9-eR_S366 | 17890 | 10317 | 57.7% |
| run_ITS_250pb_20180307 | YYT9-fR_S374 | 15645 | 9454 | 60.4% |
| run_ITS_250pb_20180307 | YYT9-gR_S382 | 20739 | 6929 | 33.4% |
| run_ITS_250pb_20180307 | YYT9-hR_S295 | 27653 | 15276 | 55.2% |
| run_ITS_250pb_20180307 | YYT9-iR_S303 | 33286 | 20049 | 60.2% |
| run_ITS_250pb_20180307 | YYT9-jR_S311 | 29805 | 12620 | 42.3% |
| run_16S_250pb_20180413 | temoin-negatif-extraction_S192 | 66455 | 64030 | 96.4% |
| run_16S_250pb_20180413 | T-POSITIF-EXRATION_S184 | 74228 | 71551 | 96.4% |
| run_16S_250pb_20180413 | T-POSITIF-EXRATION_S96 | 54346 | 51983 | 95.7% |
| run_16S_250pb_20180413 | Undetermined_S0 | 2158882 | 1558542 | 72.2% |
| run_16S_250pb_20180413 | YYM1-aT_S1 | 63006 | 58588 | 93.0% |
| run_16S_250pb_20180413 | YYM1-bT_S9 | 53308 | 49662 | 93.2% |
| run_16S_250pb_20180413 | YYM1-cT_S17 | 51859 | 46228 | 89.1% |
| run_16S_250pb_20180413 | YYM1-dT_S25 | 65568 | 61224 | 93.4% |
| run_16S_250pb_20180413 | YYM1-eT_S33 | 71906 | 65003 | 90.4% |
| run_16S_250pb_20180413 | YYM1-fT_S41 | 41870 | 38707 | 92.4% |
| run_16S_250pb_20180413 | YYM1-gT_S49 | 55657 | 50564 | 90.8% |
| run_16S_250pb_20180413 | YYM1-hT_S57 | 87445 | 83314 | 95.3% |
| run_16S_250pb_20180413 | YYM1-iT_S65 | 65865 | 62213 | 94.5% |
| run_16S_250pb_20180413 | YYM1-jT_S73 | 53853 | 49735 | 92.4% |
| run_16S_250pb_20180413 | YYM3-aT_S66 | 79376 | 75649 | 95.3% |
| run_16S_250pb_20180413 | YYM3-bT_S74 | 63090 | 59934 | 95.0% |
| run_16S_250pb_20180413 | YYM3-cT_S82 | 76302 | 72823 | 95.4% |
| run_16S_250pb_20180413 | YYM3-dT_S90 | 63762 | 60427 | 94.8% |
| run_16S_250pb_20180413 | YYM3-eT_S3 | 56408 | 52695 | 93.4% |
| run_16S_250pb_20180413 | YYM3-fT_S11 | 85261 | 80429 | 94.3% |
| run_16S_250pb_20180413 | YYM3-gT_S19 | 66986 | 63964 | 95.5% |
| run_16S_250pb_20180413 | YYM3-hT_S27 | 94632 | 90904 | 96.1% |
| run_16S_250pb_20180413 | YYM3-iT_S35 | 35047 | 33616 | 95.9% |
| run_16S_250pb_20180413 | YYM3-jT_S43 | 79442 | 76628 | 96.5% |
| run_16S_250pb_20180413 | YYM4-aT_S36 | 62880 | 58310 | 92.7% |
| run_16S_250pb_20180413 | YYM4-bT_S44 | 72037 | 68131 | 94.6% |
| run_16S_250pb_20180413 | YYM4-cT_S52 | 59901 | 57880 | 96.6% |
| run_16S_250pb_20180413 | YYM4-dT_S60 | 57472 | 54767 | 95.3% |
| run_16S_250pb_20180413 | YYM4-eT_S68 | 54174 | 49668 | 91.7% |
| run_16S_250pb_20180413 | YYM4-fT_S76 | 71343 | 67346 | 94.4% |
| run_16S_250pb_20180413 | YYM4-gT_S84 | 39919 | 37150 | 93.1% |
| run_16S_250pb_20180413 | YYM4-hT_S92 | 67598 | 64234 | 95.0% |
| run_16S_250pb_20180413 | YYM4-iT_S5 | 40190 | 36503 | 90.8% |
| run_16S_250pb_20180413 | YYM4-jT_S13 | 101747 | 98274 | 96.6% |
| run_16S_250pb_20180413 | YYM5-aT_S6 | 56060 | 51272 | 91.5% |
| run_16S_250pb_20180413 | YYM5-bT_S14 | 96833 | 92772 | 95.8% |
| run_16S_250pb_20180413 | YYM5-cT_S22 | 79601 | 75308 | 94.6% |
| run_16S_250pb_20180413 | YYM5-dT_S30 | 75556 | 72325 | 95.7% |
| run_16S_250pb_20180413 | YYM5-eT_S38 | 50286 | 47100 | 93.7% |
| run_16S_250pb_20180413 | YYM5-fT_S46 | 95664 | 90559 | 94.7% |
| run_16S_250pb_20180413 | YYM5-gT_S54 | 62268 | 59595 | 95.7% |
| run_16S_250pb_20180413 | YYM5-hT_S62 | 79145 | 74802 | 94.5% |
| run_16S_250pb_20180413 | YYM5-iT_S70 | 49629 | 45751 | 92.2% |
| run_16S_250pb_20180413 | YYM5-jT_S78 | 70384 | 66939 | 95.1% |
| run_16S_250pb_20180413 | YYM6-aT_S71 | 58941 | 55969 | 95.0% |
| run_16S_250pb_20180413 | YYM6-bT_S79 | 60082 | 55974 | 93.2% |
| run_16S_250pb_20180413 | YYM6-cT_S87 | 57662 | 54166 | 93.9% |
| run_16S_250pb_20180413 | YYM6-dT_S95 | 44442 | 41562 | 93.5% |
| run_16S_250pb_20180413 | YYM6-eT_S8 | 94458 | 90795 | 96.1% |
| run_16S_250pb_20180413 | YYM6-fT_S16 | 55995 | 52980 | 94.6% |
| run_16S_250pb_20180413 | YYM6-gT_S24 | 65587 | 62339 | 95.0% |
| run_16S_250pb_20180413 | YYM6-hT_S32 | 70806 | 66747 | 94.3% |
| run_16S_250pb_20180413 | YYM6-iT_S40 | 67637 | 64042 | 94.7% |
| run_16S_250pb_20180413 | YYM6-jT_S48 | 95577 | 91120 | 95.3% |
| run_16S_250pb_20180413 | YYM7-aT_S137 | 42149 | 39675 | 94.1% |
| run_16S_250pb_20180413 | YYM7-cT_S153 | 35388 | 33095 | 93.5% |
| run_16S_250pb_20180413 | YYM7-dT_S161 | 41684 | 39151 | 93.9% |
| run_16S_250pb_20180413 | YYM7-eT_S169 | 51049 | 48625 | 95.3% |
| run_16S_250pb_20180413 | YYM7-fT_S185 | 47288 | 45528 | 96.3% |
| run_16S_250pb_20180413 | YYM7-gT_S177 | 45797 | 42987 | 93.9% |
| run_16S_250pb_20180413 | YYM7-hT_S98 | 51707 | 49574 | 95.9% |
| run_16S_250pb_20180413 | YYM7-iT_S106 | 35773 | 33768 | 94.4% |
| run_16S_250pb_20180413 | YYM7-jT_S114 | 41163 | 38342 | 93.1% |
| run_16S_250pb_20180413 | YYM8-aT_S107 | 53304 | 50426 | 94.6% |
| run_16S_250pb_20180413 | YYM8-bT_S115 | 44412 | 41409 | 93.2% |
| run_16S_250pb_20180413 | YYM8-cT_S123 | 49166 | 45549 | 92.6% |
| run_16S_250pb_20180413 | YYM8-dT_S131 | 48124 | 45331 | 94.2% |
| run_16S_250pb_20180413 | YYM8-eT_S139 | 38732 | 35910 | 92.7% |
| run_16S_250pb_20180413 | YYM8-fT_S155 | 45180 | 42340 | 93.7% |
| run_16S_250pb_20180413 | YYM8-gT_S163 | 47467 | 45905 | 96.7% |
| run_16S_250pb_20180413 | YYM8-hT_S171 | 50271 | 46185 | 91.9% |
| run_16S_250pb_20180413 | YYM8-iT_S179 | 59454 | 55527 | 93.4% |
| run_16S_250pb_20180413 | YYM8-jT_S187 | 45260 | 43133 | 95.3% |
| run_16S_250pb_20180413 | YYM9-aT_S172 | 54701 | 51588 | 94.3% |
| run_16S_250pb_20180413 | YYM9-bT_S180 | 58293 | 56211 | 96.4% |
| run_16S_250pb_20180413 | YYM9-cT_S188 | 56038 | 53775 | 96.0% |
| run_16S_250pb_20180413 | YYM9-dT_S101 | 38545 | 36597 | 94.9% |
| run_16S_250pb_20180413 | YYM9-eT_S109 | 34666 | 32421 | 93.5% |
| run_16S_250pb_20180413 | YYM9-fT_S117 | 39767 | 37919 | 95.4% |
| run_16S_250pb_20180413 | YYM9-gT_S125 | 35153 | 33636 | 95.7% |
| run_16S_250pb_20180413 | YYM9-hT_S133 | 27747 | 26051 | 93.9% |
| run_16S_250pb_20180413 | YYM9-iT_S141 | 54112 | 51791 | 95.7% |
| run_16S_250pb_20180413 | YYM9-jT_S149 | 43993 | 40658 | 92.4% |
| run_16S_250pb_20180413 | YYT10-aT_S127 | 70986 | 68044 | 95.9% |
| run_16S_250pb_20180413 | YYT10-bT_S135 | 43457 | 39628 | 91.2% |
| run_16S_250pb_20180413 | YYT10-cT_S143 | 41689 | 38757 | 93.0% |
| run_16S_250pb_20180413 | YYT10-dT_S151 | 86780 | 83698 | 96.4% |
| run_16S_250pb_20180413 | YYT10-eT_S159 | 38645 | 35702 | 92.4% |
| run_16S_250pb_20180413 | YYT10-fT_S167 | 74716 | 69145 | 92.5% |
| run_16S_250pb_20180413 | YYT10-gT_S175 | 64780 | 60301 | 93.1% |
| run_16S_250pb_20180413 | YYT10-hT_S183 | 49479 | 44919 | 90.8% |
| run_16S_250pb_20180413 | YYT10-iT_S191 | 50679 | 46653 | 92.1% |
| run_16S_250pb_20180413 | YYT10-jT_S104 | 58333 | 53334 | 91.4% |
| run_16S_250pb_20180413 | YYT11-aT_S112 | 74932 | 72194 | 96.3% |
| run_16S_250pb_20180413 | YYT11-bT_S120 | 62153 | 59244 | 95.3% |
| run_16S_250pb_20180413 | YYT11-cT_S128 | 55153 | 51276 | 93.0% |
| run_16S_250pb_20180413 | YYT11-dT_S136 | 79084 | 76979 | 97.3% |
| run_16S_250pb_20180413 | YYT11-eT_S144 | 48782 | 46113 | 94.5% |
| run_16S_250pb_20180413 | YYT11-fT_S152 | 66557 | 62805 | 94.4% |
| run_16S_250pb_20180413 | YYT11-gT_S160 | 52268 | 49504 | 94.7% |
| run_16S_250pb_20180413 | YYT11-hT_S168 | 86025 | 73229 | 85.1% |
| run_16S_250pb_20180413 | YYT11-iT_S176 | 35098 | 32685 | 93.1% |
| run_16S_250pb_20180413 | YYT1-aT_S81 | 50027 | 47670 | 95.3% |
| run_16S_250pb_20180413 | YYT1-bT_S89 | 48156 | 45576 | 94.6% |
| run_16S_250pb_20180413 | YYT1-cT_S2 | 82294 | 77271 | 93.9% |
| run_16S_250pb_20180413 | YYT1-dT_S10 | 45910 | 42134 | 91.8% |
| run_16S_250pb_20180413 | YYT1-eT_S18 | 63086 | 60657 | 96.1% |
| run_16S_250pb_20180413 | YYT1-fT_S26 | 68926 | 64301 | 93.3% |
| run_16S_250pb_20180413 | YYT1-gT_S34 | 77179 | 73394 | 95.1% |
| run_16S_250pb_20180413 | YYT1-hT_S42 | 72064 | 67839 | 94.1% |
| run_16S_250pb_20180413 | YYT1-iT_S50 | 74453 | 69833 | 93.8% |
| run_16S_250pb_20180413 | YYT1-jT_S58 | 67044 | 63261 | 94.4% |
| run_16S_250pb_20180413 | YYT2-aT_S51 | 45753 | 42504 | 92.9% |
| run_16S_250pb_20180413 | YYT2-bT_S59 | 63756 | 60089 | 94.2% |
| run_16S_250pb_20180413 | YYT2-cT_S67 | 75331 | 71456 | 94.9% |
| run_16S_250pb_20180413 | YYT2-dT_S75 | 39804 | 36433 | 91.5% |
| run_16S_250pb_20180413 | YYT2-eT_S83 | 74007 | 71382 | 96.5% |
| run_16S_250pb_20180413 | YYT2-fT_S91 | 103669 | 95801 | 92.4% |
| run_16S_250pb_20180413 | YYT2-gT_S4 | 40735 | 37042 | 90.9% |
| run_16S_250pb_20180413 | YYT2-hT_S12 | 73530 | 68778 | 93.5% |
| run_16S_250pb_20180413 | YYT2-iT_S20 | 69024 | 66015 | 95.6% |
| run_16S_250pb_20180413 | YYT2-jT_S28 | 86669 | 82829 | 95.6% |
| run_16S_250pb_20180413 | YYT3-aT_S21 | 26563 | 24144 | 90.9% |
| run_16S_250pb_20180413 | YYT3-bT_S29 | 45575 | 42255 | 92.7% |
| run_16S_250pb_20180413 | YYT3-cT_S37 | 53482 | 50035 | 93.6% |
| run_16S_250pb_20180413 | YYT3-dT_S45 | 52043 | 47093 | 90.5% |
| run_16S_250pb_20180413 | YYT3-eT_S53 | 44793 | 41837 | 93.4% |
| run_16S_250pb_20180413 | YYT3-fT_S61 | 60072 | 56702 | 94.4% |
| run_16S_250pb_20180413 | YYT3-gT_S69 | 53902 | 51170 | 94.9% |
| run_16S_250pb_20180413 | YYT3-hT_S77 | 53149 | 49828 | 93.8% |
| run_16S_250pb_20180413 | YYT3-iT_S85 | 73383 | 69375 | 94.5% |
| run_16S_250pb_20180413 | YYT3-jT_S93 | 50870 | 47967 | 94.3% |
| run_16S_250pb_20180413 | YYT4-aT_S86 | 46807 | 44550 | 95.2% |
| run_16S_250pb_20180413 | YYT4-bT_S94 | 57876 | 54384 | 94.0% |
| run_16S_250pb_20180413 | YYT4-cT_S7 | 61338 | 57729 | 94.1% |
| run_16S_250pb_20180413 | YYT4-dT_S15 | 52616 | 47481 | 90.2% |
| run_16S_250pb_20180413 | YYT4-eT_S23 | 74864 | 69639 | 93.0% |
| run_16S_250pb_20180413 | YYT4-fT_S31 | 94926 | 88704 | 93.4% |
| run_16S_250pb_20180413 | YYT4-gT_S39 | 79153 | 74595 | 94.2% |
| run_16S_250pb_20180413 | YYT4-hT_S47 | 131694 | 126206 | 95.8% |
| run_16S_250pb_20180413 | YYT4-iT_S55 | 130609 | 126394 | 96.8% |
| run_16S_250pb_20180413 | YYT4-jT_S63 | 93481 | 88700 | 94.9% |
| run_16S_250pb_20180413 | YYT5-aT_S56 | 74806 | 70806 | 94.7% |
| run_16S_250pb_20180413 | YYT5-bT_S145 | 31576 | 29093 | 92.1% |
| run_16S_250pb_20180413 | YYT5-bT_S64 | 69437 | 65009 | 93.6% |
| run_16S_250pb_20180413 | YYT5-cT_S72 | 66288 | 62724 | 94.6% |
| run_16S_250pb_20180413 | YYT5-dT_S80 | 42074 | 39835 | 94.7% |
| run_16S_250pb_20180413 | YYT5-eT_S88 | 61694 | 57399 | 93.0% |
| run_16S_250pb_20180413 | YYT5-fT_S97 | 57124 | 54791 | 95.9% |
| run_16S_250pb_20180413 | YYT5-gT_S105 | 33331 | 30990 | 93.0% |
| run_16S_250pb_20180413 | YYT5-hT_S113 | 73107 | 70363 | 96.2% |
| run_16S_250pb_20180413 | YYT5-iT_S121 | 40414 | 38010 | 94.1% |
| run_16S_250pb_20180413 | YYT5-jT_S129 | 68570 | 65852 | 96.0% |
| run_16S_250pb_20180413 | YYT6-aT_S122 | 37345 | 34793 | 93.2% |
| run_16S_250pb_20180413 | YYT6-bT_S130 | 45365 | 42546 | 93.8% |
| run_16S_250pb_20180413 | YYT6-cT_S138 | 50690 | 48585 | 95.8% |
| run_16S_250pb_20180413 | YYT6-dT_S146 | 71154 | 68519 | 96.3% |
| run_16S_250pb_20180413 | YYT6-eT_S154 | 50087 | 47678 | 95.2% |
| run_16S_250pb_20180413 | YYT6-fT_S162 | 52747 | 48510 | 92.0% |
| run_16S_250pb_20180413 | YYT6-gT_S170 | 62893 | 60888 | 96.8% |
| run_16S_250pb_20180413 | YYT6-hT_S178 | 42369 | 40763 | 96.2% |
| run_16S_250pb_20180413 | YYT6-iT_S186 | 44201 | 42683 | 96.6% |
| run_16S_250pb_20180413 | YYT6-jT_S99 | 66380 | 63885 | 96.2% |
| run_16S_250pb_20180413 | YYT7-aT_S147 | 38392 | 35995 | 93.8% |
| run_16S_250pb_20180413 | YYT7-bT_S100 | 53439 | 50211 | 94.0% |
| run_16S_250pb_20180413 | YYT7-cT_S108 | 47277 | 44707 | 94.6% |
| run_16S_250pb_20180413 | YYT7-dT_S116 | 51541 | 49420 | 95.9% |
| run_16S_250pb_20180413 | YYT7-eT_S124 | 39785 | 37571 | 94.4% |
| run_16S_250pb_20180413 | YYT7-fT_S132 | 34878 | 32607 | 93.5% |
| run_16S_250pb_20180413 | YYT7-gT_S140 | 39288 | 38117 | 97.0% |
| run_16S_250pb_20180413 | YYT7-hT_S148 | 48794 | 46968 | 96.3% |
| run_16S_250pb_20180413 | YYT7-iT_S156 | 46374 | 44671 | 96.3% |
| run_16S_250pb_20180413 | YYT7-jT_S164 | 43129 | 40090 | 93.0% |
| run_16S_250pb_20180413 | YYT8-aT_S157 | 61423 | 58202 | 94.8% |
| run_16S_250pb_20180413 | YYT8-bT_S165 | 54194 | 50473 | 93.1% |
| run_16S_250pb_20180413 | YYT8-cT_S173 | 42599 | 39626 | 93.0% |
| run_16S_250pb_20180413 | YYT8-dT_S181 | 69515 | 66701 | 96.0% |
| run_16S_250pb_20180413 | YYT8-eT_S189 | 55658 | 53259 | 95.7% |
| run_16S_250pb_20180413 | YYT8-fT_S102 | 44777 | 42843 | 95.7% |
| run_16S_250pb_20180413 | YYT8-gT_S110 | 45855 | 42612 | 92.9% |
| run_16S_250pb_20180413 | YYT8-hT_S118 | 37688 | 35569 | 94.4% |
| run_16S_250pb_20180413 | YYT8-iT_S126 | 27957 | 25876 | 92.6% |
| run_16S_250pb_20180413 | YYT8-jT_S134 | 47974 | 46153 | 96.2% |
| run_16S_250pb_20180413 | YYT9-aT_S142 | 77790 | 75311 | 96.8% |
| run_16S_250pb_20180413 | YYT9-bT_S150 | 58308 | 54475 | 93.4% |
| run_16S_250pb_20180413 | YYT9-cT_S158 | 53734 | 51849 | 96.5% |
| run_16S_250pb_20180413 | YYT9-dT_S166 | 52647 | 49274 | 93.6% |
| run_16S_250pb_20180413 | YYT9-eT_S174 | 44494 | 42841 | 96.3% |
| run_16S_250pb_20180413 | YYT9-fT_S182 | 53796 | 51016 | 94.8% |
| run_16S_250pb_20180413 | YYT9-gT_S190 | 69004 | 65871 | 95.5% |
| run_16S_250pb_20180413 | YYT9-hT_S103 | 60257 | 57853 | 96.0% |
| run_16S_250pb_20180413 | YYT9-iT_S111 | 59761 | 57336 | 95.9% |
| run_16S_250pb_20180413 | YYT9-jT_S119 | 46185 | 44116 | 95.5% |
| run_ITS_250pb_20180413 | T-Negatif-extraction_S192 | 15274 | 13179 | 86.3% |
| run_ITS_250pb_20180413 | T-POSITIF-EXRATION_S184 | 31442 | 23398 | 74.4% |
| run_ITS_250pb_20180413 | T-POSITIF-EXRATION_S96 | 107955 | 90405 | 83.7% |
| run_ITS_250pb_20180413 | Undetermined_S0 | 2048226 | 1146054 | 56.0% |
| run_ITS_250pb_20180413 | YYM1-aT_S1 | 38265 | 28147 | 73.6% |
| run_ITS_250pb_20180413 | YYM1-bT_S9 | 77022 | 64915 | 84.3% |
| run_ITS_250pb_20180413 | YYM1-cT_S17 | 48246 | 33081 | 68.6% |
| run_ITS_250pb_20180413 | YYM1-dT_S25 | 45406 | 32911 | 72.5% |
| run_ITS_250pb_20180413 | YYM1-eT_S33 | 60380 | 49088 | 81.3% |
| run_ITS_250pb_20180413 | YYM1-fT_S41 | 35210 | 25269 | 71.8% |
| run_ITS_250pb_20180413 | YYM1-gT_S49 | 30907 | 23065 | 74.6% |
| run_ITS_250pb_20180413 | YYM1-hT_S57 | 70560 | 54964 | 77.9% |
| run_ITS_250pb_20180413 | YYM1-iT_S65 | 82932 | 62544 | 75.4% |
| run_ITS_250pb_20180413 | YYM1-jT_S73 | 66046 | 51202 | 77.5% |
| run_ITS_250pb_20180413 | YYM3-aT_S66 | 24985 | 18229 | 73.0% |
| run_ITS_250pb_20180413 | YYM3-bT_S74 | 57807 | 44827 | 77.5% |
| run_ITS_250pb_20180413 | YYM3-cT_S82 | 73268 | 56329 | 76.9% |
| run_ITS_250pb_20180413 | YYM3-dT_S90 | 35842 | 26881 | 75.0% |
| run_ITS_250pb_20180413 | YYM3-eT_S3 | 60001 | 50051 | 83.4% |
| run_ITS_250pb_20180413 | YYM3-fT_S11 | 47274 | 34118 | 72.2% |
| run_ITS_250pb_20180413 | YYM3-gT_S19 | 27565 | 21618 | 78.4% |
| run_ITS_250pb_20180413 | YYM3-hT_S27 | 31792 | 23549 | 74.1% |
| run_ITS_250pb_20180413 | YYM3-iT_S35 | 51746 | 42617 | 82.4% |
| run_ITS_250pb_20180413 | YYM3-jT_S43 | 58312 | 47182 | 80.9% |
| run_ITS_250pb_20180413 | YYM4-aT_S36 | 43709 | 31481 | 72.0% |
| run_ITS_250pb_20180413 | YYM4-bT_S44 | 37744 | 29459 | 78.0% |
| run_ITS_250pb_20180413 | YYM4-cT_S52 | 57711 | 47027 | 81.5% |
| run_ITS_250pb_20180413 | YYM4-dT_S60 | 52621 | 32131 | 61.1% |
| run_ITS_250pb_20180413 | YYM4-eT_S68 | 59691 | 43790 | 73.4% |
| run_ITS_250pb_20180413 | YYM4-fT_S76 | 39420 | 30554 | 77.5% |
| run_ITS_250pb_20180413 | YYM4-gT_S84 | 67972 | 53381 | 78.5% |
| run_ITS_250pb_20180413 | YYM4-hT_S92 | 33489 | 25346 | 75.7% |
| run_ITS_250pb_20180413 | YYM4-iT_S5 | 70786 | 56354 | 79.6% |
| run_ITS_250pb_20180413 | YYM4-jT_S13 | 49604 | 39700 | 80.0% |
| run_ITS_250pb_20180413 | YYM5-aT_S6 | 53058 | 44706 | 84.3% |
| run_ITS_250pb_20180413 | YYM5-bT_S14 | 32080 | 22654 | 70.6% |
| run_ITS_250pb_20180413 | YYM5-cT_S22 | 43732 | 35243 | 80.6% |
| run_ITS_250pb_20180413 | YYM5-dT_S30 | 37097 | 30101 | 81.1% |
| run_ITS_250pb_20180413 | YYM5-eT_S38 | 99724 | 83271 | 83.5% |
| run_ITS_250pb_20180413 | YYM5-fT_S46 | 48948 | 40535 | 82.8% |
| run_ITS_250pb_20180413 | YYM5-gT_S54 | 49304 | 38992 | 79.1% |
| run_ITS_250pb_20180413 | YYM5-hT_S62 | 25470 | 16623 | 65.3% |
| run_ITS_250pb_20180413 | YYM5-iT_S70 | 81370 | 67673 | 83.2% |
| run_ITS_250pb_20180413 | YYM5-jT_S78 | 64681 | 52145 | 80.6% |
| run_ITS_250pb_20180413 | YYM6-aT_S71 | 33286 | 26003 | 78.1% |
| run_ITS_250pb_20180413 | YYM6-bT_S79 | 36791 | 27455 | 74.6% |
| run_ITS_250pb_20180413 | YYM6-cT_S87 | 92095 | 75109 | 81.6% |
| run_ITS_250pb_20180413 | YYM6-dT_S95 | 50473 | 39701 | 78.7% |
| run_ITS_250pb_20180413 | YYM6-eT_S8 | 50452 | 43894 | 87.0% |
| run_ITS_250pb_20180413 | YYM6-fT_S16 | 61278 | 51054 | 83.3% |
| run_ITS_250pb_20180413 | YYM6-gT_S24 | 55665 | 34722 | 62.4% |
| run_ITS_250pb_20180413 | YYM6-hT_S32 | 40674 | 30424 | 74.8% |
| run_ITS_250pb_20180413 | YYM6-iT_S40 | 42500 | 35084 | 82.6% |
| run_ITS_250pb_20180413 | YYM6-jT_S48 | 71893 | 48215 | 67.1% |
| run_ITS_250pb_20180413 | YYM7-aT_S137 | 48719 | 35038 | 71.9% |
| run_ITS_250pb_20180413 | YYM7-cT_S153 | 40001 | 31612 | 79.0% |
| run_ITS_250pb_20180413 | YYM7-dT_S161 | 54103 | 35390 | 65.4% |
| run_ITS_250pb_20180413 | YYM7-eT_S169 | 76359 | 63868 | 83.6% |
| run_ITS_250pb_20180413 | YYM7-fT_S177 | 59875 | 47029 | 78.5% |
| run_ITS_250pb_20180413 | YYM7-gT_S185 | 45834 | 33024 | 72.1% |
| run_ITS_250pb_20180413 | YYM7-hT_S98 | 74228 | 52987 | 71.4% |
| run_ITS_250pb_20180413 | YYM7-iT_S106 | 47951 | 29967 | 62.5% |
| run_ITS_250pb_20180413 | YYM7-jT_S114 | 45958 | 31998 | 69.6% |
| run_ITS_250pb_20180413 | YYM8-aT_S107 | 68303 | 57842 | 84.7% |
| run_ITS_250pb_20180413 | YYM8-bT_S115 | 22472 | 14257 | 63.4% |
| run_ITS_250pb_20180413 | YYM8-cT_S123 | 33337 | 24310 | 72.9% |
| run_ITS_250pb_20180413 | YYM8-dT_S131 | 40449 | 26350 | 65.1% |
| run_ITS_250pb_20180413 | YYM8-eT_S139 | 71540 | 59149 | 82.7% |
| run_ITS_250pb_20180413 | YYM8-fT_S155 | 42940 | 33330 | 77.6% |
| run_ITS_250pb_20180413 | YYM8-gT_S163 | 39221 | 33860 | 86.3% |
| run_ITS_250pb_20180413 | YYM8-hT_S171 | 27425 | 20239 | 73.8% |
| run_ITS_250pb_20180413 | YYM8-iT_S179 | 22938 | 16561 | 72.2% |
| run_ITS_250pb_20180413 | YYM8-jT_S187 | 38056 | 24533 | 64.5% |
| run_ITS_250pb_20180413 | YYM9-aT_S172 | 35362 | 24070 | 68.1% |
| run_ITS_250pb_20180413 | YYM9-bT_S180 | 74833 | 50858 | 68.0% |
| run_ITS_250pb_20180413 | YYM9-cT_S188 | 32176 | 17594 | 54.7% |
| run_ITS_250pb_20180413 | YYM9-dT_S101 | 61336 | 46060 | 75.1% |
| run_ITS_250pb_20180413 | YYM9-eT_S109 | 30468 | 20030 | 65.7% |
| run_ITS_250pb_20180413 | YYM9-fT_S117 | 13907 | 9358 | 67.3% |
| run_ITS_250pb_20180413 | YYM9-gT_S125 | 73628 | 53354 | 72.5% |
| run_ITS_250pb_20180413 | YYM9-hT_S133 | 30129 | 19821 | 65.8% |
| run_ITS_250pb_20180413 | YYM9-iT_S141 | 57675 | 44649 | 77.4% |
| run_ITS_250pb_20180413 | YYM9-jT_S149 | 23581 | 16727 | 70.9% |
| run_ITS_250pb_20180413 | YYT10-aT_S127 | 58020 | 45604 | 78.6% |
| run_ITS_250pb_20180413 | YYT10-bT_S135 | 33689 | 26961 | 80.0% |
| run_ITS_250pb_20180413 | YYT10-cT_S143 | 31498 | 25250 | 80.2% |
| run_ITS_250pb_20180413 | YYT10-dT_S151 | 23600 | 20012 | 84.8% |
| run_ITS_250pb_20180413 | YYT10-eT_S159 | 21301 | 16585 | 77.9% |
| run_ITS_250pb_20180413 | YYT10-fT_S167 | 43281 | 34107 | 78.8% |
| run_ITS_250pb_20180413 | YYT10-gT_S175 | 23713 | 17365 | 73.2% |
| run_ITS_250pb_20180413 | YYT10-hT_S183 | 25693 | 20259 | 78.9% |
| run_ITS_250pb_20180413 | YYT10-iT_S191 | 25296 | 13618 | 53.8% |
| run_ITS_250pb_20180413 | YYT10-jT_S104 | 26174 | 19309 | 73.8% |
| run_ITS_250pb_20180413 | YYT11-aT_S112 | 40901 | 31727 | 77.6% |
| run_ITS_250pb_20180413 | YYT11-bT_S120 | 37271 | 26908 | 72.2% |
| run_ITS_250pb_20180413 | YYT11-cT_S128 | 36003 | 20851 | 57.9% |
| run_ITS_250pb_20180413 | YYT11-dT_S136 | 85518 | 58870 | 68.8% |
| run_ITS_250pb_20180413 | YYT11-eT_S144 | 38909 | 30369 | 78.1% |
| run_ITS_250pb_20180413 | YYT11-fT_S152 | 70909 | 59995 | 84.6% |
| run_ITS_250pb_20180413 | YYT11-gT_S160 | 56138 | 42982 | 76.6% |
| run_ITS_250pb_20180413 | YYT11-hT_S168 | 92178 | 32984 | 35.8% |
| run_ITS_250pb_20180413 | YYT11-iT_S176 | 50485 | 39240 | 77.7% |
| run_ITS_250pb_20180413 | YYT1-aT_S81 | 43557 | 34757 | 79.8% |
| run_ITS_250pb_20180413 | YYT1-bT_S89 | 36742 | 27595 | 75.1% |
| run_ITS_250pb_20180413 | YYT1-cT_S2 | 27837 | 20866 | 75.0% |
| run_ITS_250pb_20180413 | YYT1-dT_S10 | 29379 | 20880 | 71.1% |
| run_ITS_250pb_20180413 | YYT1-eT_S18 | 44076 | 26313 | 59.7% |
| run_ITS_250pb_20180413 | YYT1-fT_S26 | 77940 | 66522 | 85.4% |
| run_ITS_250pb_20180413 | YYT1-gT_S34 | 50955 | 39031 | 76.6% |
| run_ITS_250pb_20180413 | YYT1-hT_S42 | 19081 | 13875 | 72.7% |
| run_ITS_250pb_20180413 | YYT1-iT_S50 | 40209 | 30265 | 75.3% |
| run_ITS_250pb_20180413 | YYT1-jT_S58 | 72710 | 60308 | 82.9% |
| run_ITS_250pb_20180413 | YYT2-aT_S51 | 33256 | 22802 | 68.6% |
| run_ITS_250pb_20180413 | YYT2-bT_S59 | 15 | 8 | 53.3% |
| run_ITS_250pb_20180413 | YYT2-cT_S67 | 25185 | 19222 | 76.3% |
| run_ITS_250pb_20180413 | YYT2-dT_S75 | 62071 | 49309 | 79.4% |
| run_ITS_250pb_20180413 | YYT2-eT_S83 | 56026 | 46248 | 82.5% |
| run_ITS_250pb_20180413 | YYT2-fT_S91 | 40049 | 31383 | 78.4% |
| run_ITS_250pb_20180413 | YYT2-gT_S4 | 29382 | 22507 | 76.6% |
| run_ITS_250pb_20180413 | YYT2-hT_S12 | 39471 | 30723 | 77.8% |
| run_ITS_250pb_20180413 | YYT2-iT_S20 | 27677 | 20269 | 73.2% |
| run_ITS_250pb_20180413 | YYT2-jT_S28 | 60631 | 50045 | 82.5% |
| run_ITS_250pb_20180413 | YYT3-aT_S21 | 43105 | 34225 | 79.4% |
| run_ITS_250pb_20180413 | YYT3-bT_S29 | 22655 | 15979 | 70.5% |
| run_ITS_250pb_20180413 | YYT3-cT_S37 | 42234 | 31219 | 73.9% |
| run_ITS_250pb_20180413 | YYT3-dT_S45 | 46873 | 38616 | 82.4% |
| run_ITS_250pb_20180413 | YYT3-eT_S53 | 101502 | 88114 | 86.8% |
| run_ITS_250pb_20180413 | YYT3-fT_S61 | 24341 | 17956 | 73.8% |
| run_ITS_250pb_20180413 | YYT3-gT_S69 | 46198 | 37925 | 82.1% |
| run_ITS_250pb_20180413 | YYT3-hT_S77 | 57788 | 42217 | 73.1% |
| run_ITS_250pb_20180413 | YYT3-iT_S85 | 18105 | 14669 | 81.0% |
| run_ITS_250pb_20180413 | YYT3-jT_S93 | 22128 | 16402 | 74.1% |
| run_ITS_250pb_20180413 | YYT4-aT_S86 | 42658 | 33125 | 77.7% |
| run_ITS_250pb_20180413 | YYT4-bT_S94 | 64940 | 50612 | 77.9% |
| run_ITS_250pb_20180413 | YYT4-cT_S7 | 65827 | 46306 | 70.3% |
| run_ITS_250pb_20180413 | YYT4-dT_S15 | 39685 | 30053 | 75.7% |
| run_ITS_250pb_20180413 | YYT4-eT_S23 | 49646 | 38691 | 77.9% |
| run_ITS_250pb_20180413 | YYT4-fT_S31 | 37212 | 28970 | 77.9% |
| run_ITS_250pb_20180413 | YYT4-gT_S39 | 37112 | 27220 | 73.3% |
| run_ITS_250pb_20180413 | YYT4-hT_S47 | 50426 | 42162 | 83.6% |
| run_ITS_250pb_20180413 | YYT4-iT_S55 | 28518 | 20867 | 73.2% |
| run_ITS_250pb_20180413 | YYT4-jT_S63 | 44222 | 30383 | 68.7% |
| run_ITS_250pb_20180413 | YYT5-aT_S56 | 43088 | 35763 | 83.0% |
| run_ITS_250pb_20180413 | YYT5-bT_S145 | 12169 | 5165 | 42.4% |
| run_ITS_250pb_20180413 | YYT5-bT_S64 | 70360 | 59301 | 84.3% |
| run_ITS_250pb_20180413 | YYT5-cT_S72 | 30243 | 23288 | 77.0% |
| run_ITS_250pb_20180413 | YYT5-dT_S80 | 38716 | 31484 | 81.3% |
| run_ITS_250pb_20180413 | YYT5-eT_S88 | 58939 | 47131 | 80.0% |
| run_ITS_250pb_20180413 | YYT5-fT_S97 | 48620 | 41183 | 84.7% |
| run_ITS_250pb_20180413 | YYT5-gT_S105 | 49818 | 39216 | 78.7% |
| run_ITS_250pb_20180413 | YYT5-hT_S113 | 83028 | 70086 | 84.4% |
| run_ITS_250pb_20180413 | YYT5-iT_S121 | 57093 | 42139 | 73.8% |
| run_ITS_250pb_20180413 | YYT5-jT_S129 | 85430 | 74011 | 86.6% |
| run_ITS_250pb_20180413 | YYT6-aT_S122 | 37452 | 29689 | 79.3% |
| run_ITS_250pb_20180413 | YYT6-bT_S130 | 39003 | 31773 | 81.5% |
| run_ITS_250pb_20180413 | YYT6-cT_S138 | 90729 | 75912 | 83.7% |
| run_ITS_250pb_20180413 | YYT6-dT_S146 | 29674 | 13098 | 44.1% |
| run_ITS_250pb_20180413 | YYT6-eT_S154 | 15430 | 9362 | 60.7% |
| run_ITS_250pb_20180413 | YYT6-fT_S162 | 19528 | 13835 | 70.8% |
| run_ITS_250pb_20180413 | YYT6-gT_S170 | 25246 | 20318 | 80.5% |
| run_ITS_250pb_20180413 | YYT6-hT_S178 | 52804 | 23851 | 45.2% |
| run_ITS_250pb_20180413 | YYT6-iT_S186 | 60159 | 39937 | 66.4% |
| run_ITS_250pb_20180413 | YYT6-jT_S99 | 54382 | 44682 | 82.2% |
| run_ITS_250pb_20180413 | YYT7-aT_S147 | 23544 | 15330 | 65.1% |
| run_ITS_250pb_20180413 | YYT7-bT_S100 | 35190 | 26964 | 76.6% |
| run_ITS_250pb_20180413 | YYT7-cT_S108 | 49390 | 38578 | 78.1% |
| run_ITS_250pb_20180413 | YYT7-dT_S116 | 56105 | 46191 | 82.3% |
| run_ITS_250pb_20180413 | YYT7-eT_S124 | 21513 | 9825 | 45.7% |
| run_ITS_250pb_20180413 | YYT7-fT_S132 | 31930 | 20832 | 65.2% |
| run_ITS_250pb_20180413 | YYT7-gT_S140 | 8220 | 7346 | 89.4% |
| run_ITS_250pb_20180413 | YYT7-hT_S148 | 42735 | 28796 | 67.4% |
| run_ITS_250pb_20180413 | YYT7-iT_S156 | 40181 | 26197 | 65.2% |
| run_ITS_250pb_20180413 | YYT7-jT_S164 | 20300 | 13461 | 66.3% |
| run_ITS_250pb_20180413 | YYT8-aT_S157 | 33324 | 25460 | 76.4% |
| run_ITS_250pb_20180413 | YYT8-bT_S165 | 61989 | 45042 | 72.7% |
| run_ITS_250pb_20180413 | YYT8-cT_S173 | 34385 | 12592 | 36.6% |
| run_ITS_250pb_20180413 | YYT8-dT_S181 | 60668 | 48248 | 79.5% |
| run_ITS_250pb_20180413 | YYT8-eT_S189 | 48197 | 39891 | 82.8% |
| run_ITS_250pb_20180413 | YYT8-fT_S102 | 69371 | 59772 | 86.2% |
| run_ITS_250pb_20180413 | YYT8-gT_S110 | 47608 | 27410 | 57.6% |
| run_ITS_250pb_20180413 | YYT8-hT_S118 | 25770 | 18479 | 71.7% |
| run_ITS_250pb_20180413 | YYT8-iT_S126 | 30591 | 22225 | 72.7% |
| run_ITS_250pb_20180413 | YYT8-jT_S134 | 15431 | 9311 | 60.3% |
| run_ITS_250pb_20180413 | YYT9-aT_S142 | 75355 | 63401 | 84.1% |
| run_ITS_250pb_20180413 | YYT9-bT_S150 | 34128 | 26413 | 77.4% |
| run_ITS_250pb_20180413 | YYT9-cT_S158 | 34153 | 28906 | 84.6% |
| run_ITS_250pb_20180413 | YYT9-dT_S166 | 56690 | 46076 | 81.3% |
| run_ITS_250pb_20180413 | YYT9-eT_S174 | 51414 | 41846 | 81.4% |
| run_ITS_250pb_20180413 | YYT9-fT_S182 | 44254 | 38356 | 86.7% |
| run_ITS_250pb_20180413 | YYT9-gT_S190 | 46215 | 36792 | 79.6% |
| run_ITS_250pb_20180413 | YYT9-hT_S103 | 3132 | 2552 | 81.5% |
| run_ITS_250pb_20180413 | YYT9-iT_S111 | 53303 | 43503 | 81.6% |
| run_ITS_250pb_20180413 | YYT9-jT_S119 | 32280 | 24415 | 75.6% |

**5 16S reference sequences**

# aragorn

cd ${HOME}/Science/Databases/Silva/SSU/

RELEASE=132

URL="https://www.arb-silva.de/fileadmin/silva_databases/release_${RELEASE}/Exports"

INPUT="SILVA_${RELEASE}_SSURef_Nr99_tax_silva.fasta.gz"

# Download and check

wget -c ${URL}/${INPUT}

# Define variables and output files

OUTPUT="${INPUT/.fasta.gz/_341F_785R.fasta}"

LOG="${INPUT/.fasta.gz/_341F_785R.log}"

PRIMER_F="CCTACGGGNGGCWGCAG"

PRIMER_R="GACTACHVGGGTATCTAATCC"

ANTI_PRIMER_R="GGATTAGATACCCBDGTAGTC"

MIN_LENGTH=32

MIN_F=$(( ${#PRIMER_F} * 2 / 3 ))

MIN_R=$(( ${#PRIMER_R} * 2 / 3 ))

CUTADAPT="cutadapt --discard-untrimmed --minimum-length ${MIN_LENGTH}"

# Trim forward & reverse primers, format

zcat "${INPUT}" | sed '/^>/ ! s/U/T/g' | \

${CUTADAPT} -g "${PRIMER_F}" -O "${MIN_F}" - 2> "${LOG}" | \

${CUTADAPT} -a "${ANTI_PRIMER_R}" -O "${MIN_F}" - 2>> "${LOG}" | \

sed '/^>/ s/;/|/g ; /^>/ s/ /_/g ; /^>/ s/_/ /1' > "${OUTPUT}"

# transfert

scp -C "${OUTPUT}" kl:${HOME}/data/references/16S/

**6 16S primer clipping (add ee, dereplication and fasta conversion)**

# kl

cd ${HOME}/projects/Yunnan_Rice_2016/data/

export LC_ALL=C

module load cutadapt/latest

# Define variables, temporary files and output files

PRIMER_F="CCTACGGGNGGCWGCAG"

PRIMER_R="GACTACHVGGGTATCTAATCC"

ANTI_PRIMER_R="GGATTAGATACCCBDGTAGTC"

CUTADAPT="$(which cutadapt) --minimum-length 32 --discard-untrimmed"

VSEARCH="${HOME}/bin/vsearch/bin/vsearch --fasta_width 0 --quiet"

MIN_F=$(( ${#PRIMER_F} * 2 / 3 )) # primer match is >= 2/3 of primer length

MIN_R=$(( ${#PRIMER_R} * 2 / 3 ))

# Discard previous analyses

rm -f ./run_*/*.{fas,log,qual}

# Trim forward and anti-reverse, discard erroneous sequences, set

# aside expected error rates, dereplicate fasta

for FASTQ in ./run_16S_250pb_2018062*/*_assembled.fastq ; do

[[ -s "${FASTQ}" ]] || continue # skip empty files

SAMPLE=${FASTQ/_assembled.fastq/}

LOG="${SAMPLE}.log"

FASTA="${SAMPLE}.fas"

QUAL="${SAMPLE}.qual"

(cat "${FASTQ}" ; ${VSEARCH} --fastx_revcomp "${FASTQ}" --fastqout -) | \

${CUTADAPT} -g "${PRIMER_F}" -O "${MIN_F}" - 2> "${LOG}" | \

${CUTADAPT} -a "${ANTI_PRIMER_R}" -O "${MIN_R}" - 2>> "${LOG}" | \

${VSEARCH} \

--fastq_filter - \

--fastq_maxns 0 \

--relabel_sha1 \

--eeout \

--fastaout - 2>> "${LOG}" | \

tee >(paste - - | awk -F "[>;=\t]" '{print $2, $4, length($NF)}' | \

sort -k3,3n -k1,1d -k2,2n | \

uniq --check-chars=40 > "${QUAL}") | \

sed -r '/^>/ s/;ee=[0-9.]+// ; s/;;/;/' | \

${VSEARCH} --derep_fulllength - --sizeout --output "${FASTA}" 2>> "${LOG}"

done

# clean

find . -name "*_assembled.fastq" -delete

**7 16S Global clustering and annotation**

# kl

cd ${HOME}/projects/Yunnan_Rice_2016/

# Define variables and list files

FASTA_FILES=$(find ./data/run_16S_250pb_{20180305,20180413}/ -name "*.fas" | \

tr "\n" " " | sed 's/\n$//')

QUALITY_FILES=$(sed 's/.fas/.qual/g' <<< ${FASTA_FILES})

VSEARCH="${HOME}/bin/vsearch/bin/vsearch"

# How many samples?

N_SAMPLES=$(tr " " "\n" <<< ${FASTA_FILES} | wc -l) # 384

FOLDER=./results/${PWD/*\//}

FINAL_FASTA="${FOLDER}_16S_roots_and_stems_${N_SAMPLES}_samples.fas"

QUALITY_FILE="${FOLDER}_16S_roots_and_stems_${N_SAMPLES}_samples.qual"

DISTRIBUTION_FILE="${FOLDER}_16S_roots_and_stems_${N_SAMPLES}_samples.distr"

# Build quality file

sort -k3,3n -k1,1d -k2,2n --merge ${QUALITY_FILES} | \

uniq --check-chars=40 > "${QUALITY_FILE}" &

# Build distribution file

for f in ${FASTA_FILES} ; do

grep -H "^>" "${f}"

done | \

sed 's/.*\/// ; s/.fas:>/\t/ ; s/;size=/\t/ ; s/;$//' | \

awk 'BEGIN {FS = OFS = "\t"} {print $2, $1,$3}' > "${DISTRIBUTION_FILE}" &

# Dereplicate (vsearch)

"${VSEARCH}" \

--derep_fulllength <(cat ${FASTA_FILES}) \

--sizein \

--sizeout \

--fasta_width 0 \

--output "${FINAL_FASTA}"

# Estimate memory requirements: 14 times the size of the fasta file, in GB

MAX_MEMORY=$(awk \

-v S=$(stat --printf="%s" "${FINAL_FASTA}") \

'BEGIN {printf "%.0f\n", 14 * S / (1024*1024*1024)}')

echo $MAX_MEMORY

# compress and check number of unique sequences

bzip2 -k "${FINAL_FASTA}" &

grep -c "^>" "${FINAL_FASTA}"

# go where the scripts are

PATH_FINAL_FASTA=$(readlink -f "${FINAL_FASTA}")

(cd ${HOME}/src/

# Clustering

CLUSTERING_ID=$(sbatch \

--time=2-00:0:0 \

--mem=${MAX_MEMORY}G \

swarm_fastidious.sh "${PATH_FINAL_FASTA}")

# sort the representatives

CLUSTERING_ID=${CLUSTERING_ID##* }

[[ ${CLUSTERING_ID} ]] && \

SORTING_ID=$(sbatch \

--dependency=afterok:${CLUSTERING_ID} \

--exclude="node601" \

--time=90 \

--mem=20G \

vsearch_sort.sh \

"${PATH_FINAL_FASTA/.fas/_1f_representatives.fas}")

# chimera detection

SORTING_ID=${SORTING_ID##* }

FILTER=1

[[ ${SORTING_ID} ]] && \

sbatch \

--dependency=afterok:${SORTING_ID} \

--exclude="node601" \

--time=2-00:0:0 \

--mem=40G \

vsearch_uchime.sh \

"${PATH_FINAL_FASTA/.fas/_1f_representatives.fas}" \

"${FILTER}"

# taxonomic assignment (see https://github.com/frederic-mahe/stampa)

MARKER="SSU_16S_341F_785R"

FILTER=1

[[ ${SORTING_ID} ]] &&

sbatch \

--dependency=afterok:${SORTING_ID} \

--exclude="node601" \

--time=90 \

--mem=1G \

stampa.sh \

"${PATH_FINAL_FASTA/.fas/_1f_representatives.fas}" \

"${MARKER}" \

"${FILTER}"

)

_____________________________________________________________________

https://github.com/torognes/swarm

Mahe F, Rognes T, Quince C, de Vargas C, Dunthorn M (2014)

Swarm: robust and fast clustering method for amplicon-based studies

PeerJ 2:e593 https://doi.org/10.7717/peerj.593

Mahe F, Rognes T, Quince C, de Vargas C, Dunthorn M (2015)

Swarm v2: highly-scalable and high-resolution amplicon clustering

PeerJ 3:e1420 https://doi.org/10.7717/peerj.1420

CPU features: mmx sse sse2 sse3 ssse3 sse4.1 sse4.2 popcnt avx avx2

Database file: /dev/fd/63

Output file: ${HOME}/projects/Yunnan_Rice_2016/results/Yunnan_Rice_2016_16S_roots_and stems_384_samples_1f.swarms

Statistics file: ${HOME}/projects/Yunnan_Rice_2016/results/Yunnan_Rice_2016_16S_roots_and stems_384_samples_1f.stats

Resolution (d): 1

Threads: 16

Break OTUs: Yes

Fastidious: Yes, with boundary 3

Waiting for data... (Hit Ctrl-C and run swarm -h if you meant to read data from a file.)

Reading sequences: 100%

Indexing database: 100%

Database info: 4865843679 nt in 11739556 sequences, longest 450 nt

Hashing sequences: 100%

Building network: 100%

Clustering: 100%

Results before fastidious processing:

Number of swarms: 8194220

Largest swarm: 1035670

Counting amplicons in heavy and light swarms 100%

Heavy swarms: 214620, with 3532145 amplicons

Light swarms: 7979600, with 8207411 amplicons

Total length of amplicons in light swarms: 3409056627

Bloom filter: bits=16, m=381814342224, k=6, size=45515.8MB

Adding light swarm amplicons to Bloom filter 100%

Generated 22960471632 variants from light swarms

Checking heavy swarm amplicons against Bloom filter 100%

Heavy variants: 9812874617

Got 7930709 graft candidates

Grafting light swarms on heavy swarms 100%

Made 1443165 grafts

Writing swarms: 100%

Writing seeds: 100%

Writing structure: 100%

Writing stats: 100%

Number of swarms: 6751055

Largest swarm: 1388555

Max generations: 52

Found 318193 (76.4%) chimeras, 86621 (20.8%) non-chimeras,

and 11735 (2.8%) borderline sequences in 416549 unique sequences.

Taking abundance information into account, this corresponds to

3264163 (22.7%) chimeras, 10939553 (76.0%) non-chimeras,

and 186189 (1.3%) borderline sequences in 14389905 total sequences.

**8 16S OTU table**

# kl

cd ${HOME}/projects/Yunnan_Rice_2016/results/

module load python/2.7

## Yunnan ITS2 (roots and stems)

FASTA="Yunnan_Rice_2016_16S_roots_and_stems_384_samples.fas"

SRC="${HOME}/src"

SCRIPT="${SRC}/OTU_contingency_table_filtered3.py"

STATS="${FASTA/.fas/_1f.stats}"

SWARMS="${FASTA/.fas/_1f.swarms}"

UCHIME="${FASTA/.fas/_1f_representatives.uchime}"

ASSIGNMENTS="${FASTA/.fas/_1f_representatives.results}"

REPRESENTATIVES="${FASTA/.fas/_1f_representatives.fas}"

QUALITY="${FASTA/.fas/.qual}"

DISTRIBUTION="${FASTA/.fas/.distr}"

OTU_TABLE="${FASTA/.fas/.OTU.filtered.table}"

ERROR="${FASTA/.fas/.OTU.filtered.table.error}"

# build OTU table

python \

"${SCRIPT}" \

"${REPRESENTATIVES}" \

"${STATS}" \

"${SWARMS}" \

"${UCHIME}" \

"${QUALITY}" \

"${ASSIGNMENTS}" \

"${DISTRIBUTION}" | \

sed 's/#//g' > "${OTU_TABLE}" 2> "${ERROR}"

# clean

[[ -s "${ERROR}" ]] || rm -f "${ERROR}"

**9 ITS2 reference sequences**

Unite's [repository](https://unite.ut.ee/repository.php) offers several versions and several flavors. The different flavors correspond to different clustering levels, expert annotation, singleton handling and pipeline (QIIME, Mothur, Blast, etc).

# kl

cd ${HOME}/data/references/ITS/Unite/

module load cutadapt/latest

UNITE_FASTA="sh_general_release_dynamic_all_02.02.2019.fasta"

DATE="20190202"

MIN_LENGTH=32

CUTADAPT="$(which cutadapt) --minimum-length ${MIN_LENGTH}"

PRIMER_F="GTGAATCATCGAATCTTTGAA"

PRIMER_F_NAME="ITS86F"

PRIMER_R="GCATATCAATAAGCGGAGGA" ## ITS4

MIN_F=$(( ${#PRIMER_F} * 2 / 3 ))

MIN_R=$(( ${#PRIMER_R} * 2 / 3 ))

FINAL_FASTA="unite_${PRIMER_F_NAME}_${DATE}.fasta"

LOG=${FINAL_FASTA/.fasta/.log}

# eliminate non-ASCII chars (in one fasta entry)

sed 's/×//g' "${UNITE_FASTA}" | \

${CUTADAPT} --discard-untrimmed -g "${PRIMER_F}" -O "${MIN_F}" - 2> "${LOG}" | \

${CUTADAPT} -a "${PRIMER_R}" -O "${MIN_R}" - 2>> "${LOG}" | \

sed -r '/^>/ s/\|(refs|reps|refs_singleton)\|/ / ; s/;/|/g' > "${FINAL_FASTA}"

**10 ITS2 primer clipping (add ee, dereplication and fasta conversion)**

# kl

cd ${HOME}/projects/Yunnan_Rice_2016/data/

export LC_ALL=C

module load cutadapt/latest

# Define variables, temporary files and output files

PRIMER_F="GTGAATCATCGAATCTTTGAA"

PRIMER_R="TCCTCCGCTTATTGATATGC"

ANTI_PRIMER_R="GCATATCAATAAGCGGAGGA"

CUTADAPT="$(which cutadapt) --minimum-length 32 --discard-untrimmed"

VSEARCH="${HOME}/bin/vsearch/bin/vsearch --fasta_width 0 --quiet"

MIN_F=$(( ${#PRIMER_F} * 2 / 3 )) # primer match is >= 2/3 of primer length

MIN_R=$(( ${#PRIMER_R} * 2 / 3 ))

# Trim forward and anti-reverse, discard erroneous sequences, set

# aside expected error rates, dereplicate fasta

for FASTQ in ./run_ITS_250pb_{20180307,20180413}/*_assembled.fastq ; do

SAMPLE=${FASTQ/_assembled.fastq/}

LOG="${SAMPLE}.log"

FASTA="${SAMPLE}.fas"

QUAL="${SAMPLE}.qual"

(cat "${FASTQ}" ; ${VSEARCH} --fastx_revcomp "${FASTQ}" --fastqout -) | \

${CUTADAPT} -g "${PRIMER_F}" -O "${MIN_F}" - 2> "${LOG}" | \

${CUTADAPT} -a "${ANTI_PRIMER_R}" -O "${MIN_R}" - 2>> "${LOG}" | \

${VSEARCH} \

--fastq_filter - \

--fastq_maxns 0 \

--relabel_sha1 \

--eeout \

--fastaout - 2>> "${LOG}" | \

tee >(paste - - | awk -F "[>;=\t]" '{print $2, $4, length($NF)}' | \

sort -k3,3n -k1,1d -k2,2n | \

uniq --check-chars=40 > "${QUAL}") | \

${VSEARCH} --derep_fulllength - --sizeout --output - 2>> "${LOG}" | \

sed -r '/^>/ s/;ee=[0-9.]+// ; s/;;/;/' > "${FASTA}"

done

# clean

find . -name "*_assembled.fastq" -delete

**11 ITS2 Global clustering and annotation**

# kl

cd ${HOME}/projects/Yunnan_Rice_2016/

# Define variables and list files

FASTA_FILES=$(find ./data/run_ITS_250pb_{20180307,20180413}/ -name "*.fas" | \

tr "\n" " " | sed 's/\n$//')

QUALITY_FILES=$(sed 's/.fas/.qual/g' <<< ${FASTA_FILES})

VSEARCH="${HOME}/bin/vsearch/bin/vsearch"

# How many samples?

N_SAMPLES=$(tr " " "\n" <<< ${FASTA_FILES} | wc -l) # 384

FOLDER=./results/${PWD/*\//}

FINAL_FASTA="${FOLDER}_ITS2_roots_and_stems_${N_SAMPLES}_samples.fas"

QUALITY_FILE="${FOLDER}_ITS2_roots_and_stems_${N_SAMPLES}_samples.qual"

DISTRIBUTION_FILE="${FOLDER}_ITS2_roots_and_stems_${N_SAMPLES}_samples.distr"

# Build quality file

sort -k3,3n -k1,1d -k2,2n --merge ${QUALITY_FILES} | \

uniq --check-chars=40 > "${QUALITY_FILE}" &

# Build distribution file

for f in ${FASTA_FILES} ; do

grep -H "^>" "${f}"

done | \

sed 's/.*\/// ; s/.fas:>/\t/ ; s/;size=/\t/ ; s/;$//' | \

awk 'BEGIN {FS = OFS = "\t"} {print $2, $1,$3}' > "${DISTRIBUTION_FILE}" &

# Dereplicate (vsearch)

"${VSEARCH}" \

--derep_fulllength <(cat ${FASTA_FILES}) \

--sizein \

--sizeout \

--fasta_width 0 \

--output "${FINAL_FASTA}"

# Estimate memory requirements: 14 times the size of the fasta file, in GB

MAX_MEMORY=$(awk \

-v S=$(stat --printf="%s" "${FINAL_FASTA}") \

'BEGIN {printf "%.0f\n", 14 * S / (1024*1024*1024)}')

echo $MAX_MEMORY

# compress and check number of unique sequences

bzip2 -k "${FINAL_FASTA}" &

grep -c "^>" "${FINAL_FASTA}"

# go where the scripts are

PATH_FINAL_FASTA=$(readlink -f "${FINAL_FASTA}")

(cd ${HOME}/src/

# Clustering

CLUSTERING_ID=$(sbatch \

--time=2-00:0:0 \

--mem=${MAX_MEMORY}G \

swarm_fastidious.sh "${PATH_FINAL_FASTA}")

# sort the representatives

CLUSTERING_ID=${CLUSTERING_ID##* }

[[ ${CLUSTERING_ID} ]] && \

SORTING_ID=$(sbatch \

--dependency=afterok:${CLUSTERING_ID} \

--exclude="node601" \

--time=90 \

--mem=20G \

vsearch_sort.sh \

"${PATH_FINAL_FASTA/.fas/_1f_representatives.fas}")

# chimera detection

SORTING_ID=${SORTING_ID##* }

FILTER=1

[[ ${SORTING_ID} ]] && \

sbatch \

--dependency=afterok:${SORTING_ID} \

--exclude="node601" \

--time=2-00:0:0 \

--mem=40G \

vsearch_uchime.sh \

"${PATH_FINAL_FASTA/.fas/_1f_representatives.fas}" \

"${FILTER}"

# taxonomic assignment (see https://github.com/frederic-mahe/stampa)

MARKER="ITS86F_ITS4"

FILTER=1

[[ ${SORTING_ID} ]] &&

sbatch \

--dependency=afterok:${SORTING_ID} \

--exclude="node601" \

--time=90 \

--mem=1G \

stampa.sh \

"${PATH_FINAL_FASTA/.fas/_1f_representatives.fas}" \

"${MARKER}" \

"${FILTER}"

)

______________________________________________________________________

https://github.com/torognes/swarm

Mahe F, Rognes T, Quince C, de Vargas C, Dunthorn M (2014)

Swarm: robust and fast clustering method for amplicon-based studies

PeerJ 2:e593 https://doi.org/10.7717/peerj.593

Mahe F, Rognes T, Quince C, de Vargas C, Dunthorn M (2015)

Swarm v2: highly-scalable and high-resolution amplicon clustering

PeerJ 3:e1420 https://doi.org/10.7717/peerj.1420

CPU features: mmx sse sse2 sse3 ssse3 sse4.1 sse4.2 popcnt avx avx2

Database file: /dev/fd/63

Output file: ${HOME}/projects/Yunnan_Rice_2016/results/Yunnan_Rice_2016_ITS2_roots_and stems_384_samples_1f.swarms

Statistics file: ${HOME}/projects/Yunnan_Rice_2016/results/Yunnan_Rice_2016_ITS2_roots_and stems_384_samples_1f.stats

Resolution (d): 1

Threads: 16

Break OTUs: Yes

Fastidious: Yes, with boundary 3

Waiting for data... (Hit Ctrl-C and run swarm -h if you meant to read data from a file.)

Reading sequences: 100%

Indexing database: 100%

Database info: 551619030 nt in 1792605 sequences, longest 448 nt

Hashing sequences: 100%

Building network: 100%

Clustering: 100%

Results before fastidious processing:

Number of swarms: 595516

Largest swarm: 113785

Counting amplicons in heavy and light swarms 100%

Heavy swarms: 20741, with 1211440 amplicons

Light swarms: 574775, with 581165 amplicons

Total length of amplicons in light swarms: 194264827

Bloom filter: bits=16, m=21757660624, k=6, size=2593.7MB

Adding light swarm amplicons to Bloom filter 100%

Generated 1306716178 variants from light swarms

Checking heavy swarm amplicons against Bloom filter 100%

Heavy variants: 2405667561

Got 1418900 graft candidates

Grafting light swarms on heavy swarms 100%

Made 252365 grafts

Writing swarms: 100%

Writing seeds: 100%

Writing structure: 100%

Writing stats: 100%

Number of swarms: 343151

Largest swarm: 146582

Max generations: 16

Found 12789 (50.7%) chimeras, 11972 (47.5%) non-chimeras,

and 455 (1.8%) borderline sequences in 25216 unique sequences.

Taking abundance information into account, this corresponds to

339318 (3.2%) chimeras, 10137724 (96.5%) non-chimeras,

and 27380 (0.3%) borderline sequences in 10504422 total sequences.

**12 ITS2 OTU table**

# kl

cd ${HOME}/projects/Yunnan_Rice_2016/results/

module load python/2.7

## Yunnan ITS2 (roots and stems)

FASTA="Yunnan_Rice_2016_ITS2_roots_and_stems_384_samples.fas"

SRC="${HOME}/src"

SCRIPT="${SRC}/OTU_contingency_table_filtered3.py"

STATS="${FASTA/.fas/_1f.stats}"

SWARMS="${FASTA/.fas/_1f.swarms}"

UCHIME="${FASTA/.fas/_1f_representatives.uchime}"

ASSIGNMENTS="${FASTA/.fas/_1f_representatives.results}"

REPRESENTATIVES="${FASTA/.fas/_1f_representatives.fas}"

QUALITY="${FASTA/.fas/.qual}"

DISTRIBUTION="${FASTA/.fas/.distr}"

OTU_TABLE="${FASTA/.fas/.OTU.filtered.table}"

ERROR="${FASTA/.fas/.OTU.filtered.table.error}"

# build OTU table

python \

"${SCRIPT}" \

"${REPRESENTATIVES}" \

"${STATS}" \

"${SWARMS}" \

"${UCHIME}" \

"${QUALITY}" \

"${ASSIGNMENTS}" \

"${DISTRIBUTION}" | \

sed 's/#//g' > "${OTU_TABLE}" 2> "${ERROR}"

# clean

[[ -s "${ERROR}" ]] || rm -f "${ERROR}"

Date: 2020-05-08 ven. 00:00

Author: frédéric

Created: 2020-05-08 ven. 17:00

[Validate](http://validator.w3.org/check?uri=referer)
