# Supplementary material for: Heterogeneity of the rice microbial community of the Chinese centuries‐old Honghe Hani rice terraces system
Source: Environ Microbiol. 2020 Jul 7;22(8):3429–45. doi: 10.1111/1462-2920.15114 (PMC7497281; doi:10.1111/1462-2920.15114)
Supplement: Supplementary file 10 — Table S1 Abundance of reads and OTUs in bacterial and fungal community data sets for stem and root samples through the different steps of the bio‐informatics treatments. [file EMI-22-3429-s010.docx]

**Table S1**. Abundance of reads and OTUs in bacterial and fungal community data sets for stem and root samples through the different steps of the bio-informatics treatments.

|  | **Stem bacterial community data** | | **Root bacterial community data** | | | **Stem fungal community data** | | | **Root fungal community data** | | |
| --- | --- | --- | --- | --- | --- | --- | --- | --- | --- | --- | --- |
|  | number of reads | number of OTUs | number of reads | number of OTUs | number of reads | | number of OTUs | number of reads | | number of OTUs |  |
| OTU table production | 6 000 025 | 2 098 | 3 255 114 | 3 568 | 6 210 007 | | 4 795 | 3 436 431 | | 3 712 |  |
| Contaminant sequences removal | 2 037 908 | 1 873 | 934 229 | 3 223 | 6 155 784 | | 4 783 | 3 371 906 | | 3 703 |  |
| No hit removal | 1 950 809 | 965 | 913 221 | 2 597 | 6 153 949 | | 4 738 | 3 369 332 | | 3 635 |  |
| Chloroplast and mitochondrial DNA removal | 1 204 202 | 899 | 391 243 | 899 | 6 153 949 | | 4 738 | 3 369 332 | | 3 635 |  |
| OTUs threshold at 50% prevalence and samples with low sequencing depth removal | 564 701 | 133 | 776 899 | 404 | 4 767 120 | | 250 | 1 608 894 | | 265 |  |
| Rarefaction | 92 400 | 91 | 120 666 | 325 | 21 750 | | 110 | 30 858 | | 105 |  |
