# Supplementary material for: Heterogeneity of the rice microbial community of the Chinese centuries‐old Honghe Hani rice terraces system
Source: Environ Microbiol. 2020 Jul 7;22(8):3429–45. doi: 10.1111/1462-2920.15114 (PMC7497281; doi:10.1111/1462-2920.15114)
Supplement: Supplementary file 13 — Table S4 Pairwise permutational multivariate analysis of variance (PERMANOVA) results for bacterial and fungal communities for both stem and root samples using unweighted UniFrac distance values (10,000 permutations), R2 denotes the proportion of variance that could be explained by the grouping. P. adjusted corresponds to the Bonferroni correction applied to adjust the P‐value for multiple comparisons within each group. Sig significance level *P. adjusted <0.05, **P. adjusted <0.01 and NS not significant. [file EMI-22-3429-s013.docx]

**Table S4**. Pairwise permutational multivariate analysis of variance (PERMANOVA) results for bacterial and fungal communities for both stem and root samples using unweighted UniFrac distance values (10,000 permutations), R^2^ denotes the proportion of variance that could be explained by the grouping. P.adjusted corresponds to the Bonferroni correction applied to adjust the P-value for multiple comparisons within each group. sig significance level * P.adjusted <0.05, ** P.adjusted <0.01 and NS not significant.

|  | 16S roots | | | | | | | 16S stems | | | | | | | | | ITS roots | | | | | | | | | | ITS stems | | | | | | |  |
| --- | --- | --- | --- | --- | --- | --- | --- | --- | --- | --- | --- | --- | --- | --- | --- | --- | --- | --- | --- | --- | --- | --- | --- | --- | --- | --- | --- | --- | --- | --- | --- | --- | --- | --- |
|  |  | R² | P.value | P. adjusted | | sig |  | | R² | | P.value | | P. adjusted | | sig | |  | | R² | | P.value | | P. adjusted | | sig | | R2 | P.value | | P. adjusted | | sig | |  |
| Modern 1 vs Modern 2 |  | 0.03 | 0.0256 | 0.384 | NS | |  | | | 0.06 | | 0.0266 | | 0.399 | | NS | |  | | 0.10 | | 0.0001 | | 0.002 | | ** | 0.05 | | 4.00E-04 | | 0.006 | | ** | |
| Modern 1 vs Landrace 3 |  | 0.12 | 1.00E-04 | 0.002 | ** | |  | | | 0.11 | | 0.0008 | | 0.012 | | * | |  | | 0.16 | | 0.0001 | | 0.002 | | ** | 0.11 | | 1.00E-04 | | 0.002 | | ** | |
| Modern 1 vs Landrace 1 |  | 0.06 | 1.00E-04 | 0.002 | ** | |  | | | 0.03 | | 0.2616 | | 1.000 | | NS | |  | | 0.10 | | 0.0001 | | 0.002 | | ** | 0.06 | | 1.00E-04 | | 0.002 | | ** | |
| Modern 1 vs Landrace 2 |  | 0.11 | 1.00E-04 | 0.002 | ** | |  | | | 0.08 | | 0.0191 | | 0.287 | | NS | |  | | 0.11 | | 0.0001 | | 0.002 | | ** | 0.10 | | 1.00E-04 | | 0.002 | | ** | |
| Modern 1 vs Landrace 4 |  | 0.10 | 1.00E-04 | 0.002 | ** | |  | | | 0.20 | | 0.0001 | | 0.002 | | ** | |  | | 0.18 | | 0.0001 | | 0.002 | | ** | 0.07 | | 1.00E-04 | | 0.002 | | ** | |
| Modern 2 vs Landrace 3 |  | 0.14 | 1.00E-04 | 0.002 | ** | |  | | | 0.15 | | 1.00E-04 | | 0.002 | | ** | |  | | 0.24 | | 0.0001 | | 0.002 | | ** | 0.16 | | 1.00E-04 | | 0.002 | | ** | |
| Modern 2 vs Landrace 1 |  | 0.06 | 0.0088 | 0.132 | NS | |  | | | 0.11 | | 0.0015 | | 0.023 | | * | |  | | 0.13 | | 0.0001 | | 0.002 | | ** | 0.07 | | 1.00E-04 | | 0.002 | | ** | |
| Modern 2 vs Landrace 2 |  | 0.15 | 1.00E-04 | 0.002 | ** | |  | | | 0.16 | | 4.00E-04 | | 0.006 | | ** | |  | | 0.19 | | 0.0002 | | 0.003 | | ** | 0.16 | | 1.00E-04 | | 0.002 | | ** | |
| Modern 2 vs Landrace 4 |  | 0.12 | 2.00E-04 | 0.003 | ** | |  | | | 0.38 | | 1.00E-04 | | 0.002 | | ** | |  | | 0.17 | | 0.0001 | | 0.002 | | ** | 0.09 | | 1.00E-04 | | 0.002 | | ** | |
| Landrace 3 vs Landrace 4 |  | 0.24 | 1.00E-04 | 0.002 | ** | |  | | | 0.50 | | 1.00E-04 | | 0.002 | | ** | |  | | 0.35 | | 1.00E-04 | | 0.002 | | ** | 0.18 | | 1.00E-04 | | 0.002 | | ** | |
| Landrace 1 vs Landrace 2 |  | 0.16 | 1.00E-04 | 0.002 | ** | |  | | | 0.08 | | 0.055 | | 0.825 | | NS | |  | | 0.18 | | 0.0001 | | 0.002 | | ** | 0.13 | | 1.00E-04 | | 0.002 | | ** | |
| Landrace 1 vs Landrace 4 |  | 0.09 | 2.00E-04 | 0.003 | ** | |  | | | 0.15 | | 0.0025 | | 0.038 | | * | |  | | 0.25 | | 0.0001 | | 0.002 | | ** | 0.07 | | 0.0005 | | 0.008 | | ** | |
| Landrace 2 vs Landrace 4 |  | 0.25 | 1.00E-04 | 0.002 | ** | |  | | | 0.20 | | 0.0014 | | 0.021 | | * | |  | | 0.32 | | 0.0001 | | 0.002 | | ** | 0.14 | | 2.00E-04 | | 0.003 | | ** | |
