# Supplementary material for: Heterogeneity of the rice microbial community of the Chinese centuries‐old Honghe Hani rice terraces system
Source: Environ Microbiol. 2020 Jul 7;22(8):3429–45. doi: 10.1111/1462-2920.15114 (PMC7497281; doi:10.1111/1462-2920.15114)
Supplement: Supplementary file 16 — Table S7 Detection of phylogenetic signal in root‐associated and stem‐associated microbial communities. Mantel statistic based on Pearson's product–moment correlation to assess the correlation between unweighted UniFrac distances matrix of microbial community dissimilarities and the rice genetic distances (10,000 permutations), R2 denotes the proportions of variances that could be explained by the grouping. [file EMI-22-3429-s016.docx]

**Table S7**. Detection of phylogenetic signal in root-associated and stem-associated microbial communities. Mantel statistic based on Pearson's product-moment correlation to assess the correlation between unweighted UniFrac distances matrix of microbial community dissimilarities and the rice genetic distances (1000 permutations), R^2^ denotes the proportions of variances that could be explained by the grouping.

|  | **R²** | **Pr (< F)** |
| --- | --- | --- |
| Stems bacterial communities | **0.05** | **0.0001** |
| Roots bacterial communities | **0.15** | **0.0101** |
| Stems fungal communities | **0.16** | **0.0001** |
| Roots fungal communities | **0.15** | **0.0001** |
