# Supplementary material for: Heterogeneity of the rice microbial community of the Chinese centuries‐old Honghe Hani rice terraces system
Source: Environ Microbiol. 2020 Jul 7;22(8):3429–45. doi: 10.1111/1462-2920.15114 (PMC7497281; doi:10.1111/1462-2920.15114)
Supplement: Supplementary file 17 — Table S8 List of the taxa that were present in more than 80% of the plant samples of each rice genetic group. [file EMI-22-3429-s017.docx]

**Table S8**. List of the taxa that were present in more than 80% of the plant samples of each rice genetic group.

|  | **Taxa** | **Prevalence** | **Relative Abundance** |
| --- | --- | --- | --- |
| **Stems** |  |  |  |
|  | - **Modern rice varieties**  **Bacterial taxa**  None OTU  **Fungal taxa**  Ascomycota_;Dothideomycetes_;Capnodiales_;Cladosporiaceae_;NA_;NA_;3  Ascomycota_;Dothideomycetes_;Pleosporales_;NA_;NA_;NA_; 5 | 0.96  0.87 | 18.5%  10.3% |
|  | - **HHRTS landraces**   **Bacterial taxa**  Proteobacteria_;Gammaproteobacteria_;Enterobacterales_;Erwiniaceae_;Pantoea_;NA_;3  **Fungal taxa**  Ascomycota_;Dothideomycetes_;Capnodiales_;Cladosporiaceae_;NA_;NA_;3  Ascomycota_;Dothideomycetes_;Pleosporales_;NA_;NA_;NA_;5 | 0.84  0.91  0.81 | 19.5%  22.3%  13.6% |
| **Roots** |  |  |  |
|  | - **Modern rice varieties**  **Bacterial taxa**  Myxococcota_;Myxococcia_;Myxococcales_;Myxococcaceae_;P3OB-42_;NA_;6  Myxococcota_;Myxococcia_;Myxococcales_;Myxococcaceae_;P3OB-42_;NA_;74  Myxococcota_;Myxococcia_;Myxococcales_;Myxococcaceae_;P3OB-42_;NA_;34  Myxococcota_;Myxococcia_;Myxococcales_;Myxococcaceae_;P3OB-42_;NA_;25  Myxococcota_;Myxococcia_;Myxococcales_;Myxococcaceae_;P3OB-42_;NA_;9  **Fungal taxa**  Ascomycota_;Dothideomycetes_;Capnodiales_;Cladosporiaceae_;NA_;Cladosporiaceae_sp_;3 | 1.00  0.88  0.88  0.83  0.97  0.80 | 26.4%  0.7%  3.0%  3.9%  13.5%  10.0% |
|  | - **HHRTS landraces**   **Bacterial taxa**  Myxococcota_;Myxococcia_;Myxococcales_;Myxococcaceae_;P3OB-42_;NA_;6  Myxococcota_;Myxococcia_;Myxococcales_;Myxococcaceae_;P3OB-42_;NA_;9  **Fungal taxa**  Ascomycota_;Dothideomycetes_;Capnodiales_;Cladosporiaceae_;NA_;NA_;3 | 0.94  0.84  0.86 | 18.7%  8.1%  14.3% |
