# Supplementary material for: Heterogeneity of the rice microbial community of the Chinese centuries‐old Honghe Hani rice terraces system
Source: Environ Microbiol. 2020 Jul 7;22(8):3429–45. doi: 10.1111/1462-2920.15114 (PMC7497281; doi:10.1111/1462-2920.15114)
Supplement: Supplementary file 18 — Table S9 Permutational multivariate analysis of variance (PERMANOVA) results for the influence of infection of SRBSDV on bacterial and fungal composition using UniFrac distance values (10,000 permutations), R2 denotes the proportions of variances that could be explained by the grouping. [file EMI-22-3429-s018.docx]

**Table S9**. Permutational multivariate analysis of variance (PERMANOVA) results for the influence of infection of SRBSDV on bacterial and fungal composition using UniFrac distance values (10000 permutations), R^2^ denotes the proportions of variances that could be explained by the grouping.

|  | F model | R² | Pr (< F) |
| --- | --- | --- | --- |
| Stems bacterial communities | 0.79 | 0.01 | 0.5497 |
| Roots bacterial communities | 1.24 | 0.01 | 0.1874 |
| Stems fungal communities | 7.64 | 0.01 | 0.3245 |
| Roots fungal communities | 0.89 | 0.01 | 0.5456 |
